# R-flurbiprofen attenuates experimental autoimmune encephalomyelitis in mice

**DOI:** 10.15252/emmm.201404168

**Published:** 2014-09-30

**Authors:** Katja Schmitz, Natasja de Bruin, Philipp Bishay, Julia Männich, Annett Häussler, Christine Altmann, Nerea Ferreirós, Jörn Lötsch, Alfred Ultsch, Michael J Parnham, Gerd Geisslinger, Irmgard Tegeder

**Affiliations:** 1 Institute of Clinical Pharmacology, Goethe-University HospitalFrankfurt am Main, Germany; 2 Fraunhofer Institute of Molecular Biology and Applied Ecology, Project Group Translational Medicine and Pharmacology (IME-TMP)Frankfurt am Main, Germany; 3 DataBionics Research Group, University of MarburgMarburg, Germany

**Keywords:** endocannabinoids, multiple sclerosis, optic neuritis, pain, regulatory T cells

## Abstract

R-flurbiprofen is the non-cyclooxygenase inhibiting R-enantiomer of the non-steroidal anti-inflammatory drug flurbiprofen, which was assessed as a remedy for Alzheimer's disease. Because of its anti-inflammatory, endocannabinoid-modulating and antioxidative properties, combined with low toxicity, the present study assessed R-flurbiprofen in experimental autoimmune encephalomyelitis (EAE) models of multiple sclerosis in mice. Oral R-flurbiprofen prevented and attenuated primary progressive EAE in C57BL6/J mice and relapsing-remitting EAE in SJL mice, even if the treatment was initiated on or after the first flare of the disease. R-flurbiprofen reduced immune cell infiltration and microglia activation and inflammation in the spinal cord, brain and optic nerve and attenuated myelin destruction and EAE-evoked hyperalgesia. R-flurbiprofen treatment increased CD4^+^CD25^+^FoxP3^+^ regulatory T cells, CTLA4^+^ inhibitory T cells and interleukin-10, whereas the EAE-evoked upregulation of pro-inflammatory genes in the spinal cord was strongly reduced. The effects were associated with an increase of plasma and cortical endocannabinoids but decreased spinal prostaglandins, the latter likely due to R to S inversion. The promising results suggest potential efficacy of R-flurbiprofen in human MS, and its low toxicity may justify a clinical trial.

## Introduction

R-flurbiprofen, also known as tarenflurbil, is a 2-aryl propionic acid, which has been marketed for the treatment of pain and inflammation together with its S-enantiomer as a racemate. S-flurbiprofen is a potent inhibitor of cyclooxygenases (COX) resulting in non-specific inhibition of prostaglandin synthesis. R-flurbiprofen itself is at least 100-fold less active as COX-inhibitor (Geisslinger *et al*, 2000) and has been considered as the inactive constituent of the racemate. However, R-flurbiprofen reduces pain and inflammation in humans (Lotsch *et al*, 1995) and Sprague–Dawley rats (Bishay *et al*, 2010; Geisslinger *et al*, 1993; Tegeder *et al*, 2001), species which do not essentially invert R- to S-flurbiprofen. R-flurbiprofen is almost free of the side-effects typical of classical NSAIDs, such as gastrointestinal or renal toxicity (Holzer *et al*, 2001). Its mechanisms of action involve inhibition of the transcription factors NF-κB and AP1 (Tegeder *et al*, 2001), inhibition of acid sensing ion channels (ASIC1) (Mishra *et al*, 2010) and inhibition of endocannabinoid hydrolysis or oxidation (Bishay *et al*, 2010; Duggan *et al*, 2011) with a resulting general facilitation of actions at cannabinoid CB1 and CB2 receptors on neurons, microglia and peripheral immune cells (Bishay *et al*, 2010). In particular, R-flurbiprofen treatment after peripheral nerve injury prevents microglia from adopting a phagocyte-like activated phenotype and thereby blocks the neuroinflammatory component of neuropathic pain (Bishay *et al*, 2010). Either as a consequence of the pro-endocannabinoid effects or possibly directly, R-flurbiprofen increases activation of the peroxisome proliferator activated receptors, PPARγ and PPARα, contributing to anti-inflammatory and neuroprotective effects in the brain (Bernardo *et al*, 2005, 2006; Bishay *et al*, 2010). And recently, racemic flurbiprofen was also found to prevent ER stress and leptin resistance and was suggested to provide anti-obesity activity (Hosoi *et al*, 2014).

Because of the beneficial anti-inflammatory efficacy and essential lack of toxicity, R-flurbiprofen has been evaluated as a potential remedy in Alzheimer's disease with some success in clinical trials (Wilcock *et al*, 2008). The rationale for these studies was a γ-secretase-modulating effect resulting in a weak inhibition of the generation of the amyloid β1–42 peptide (Imbimbo, 2008). Consequently, long-term treatment with R-flurbiprofen of amyloid peptide-overexpressing mice, before appearance of cognitive dysfunction, resulted in a reduction of brain plaque formation, Aβ42 deposition and improvement of spatial learning behavior (Kukar *et al*, 2007), and prevention of mitochondrial calcium overload evoked by Aβ-oligomers (Sanz-Blasco *et al*, 2008). Under oxidative stress conditions, it reduced nitrite/nitrate levels and lipid peroxidation in the brain and enhanced the release of the anti-inflammatory interleukin-10 (Lopez-Villodres *et al*, 2011, 2012). The beneficial effects of the drug may be partly lost in the aging brain (Bishay *et al*, 2013), and ultimately, its development was discontinued for Alzheimer's disease because efficacy in clinical phase III trials was insufficient (Green *et al*, 2009). This also holds true for another indication, prostate cancer, for which it was studied based on pre-clinical studies in the TRAMP mouse prostate cancer model, in which R-flurbiprofen was able to reduce the incidence of prostate tumors and metastases (Wechter *et al*, 2000).

Hence, R-flurbiprofen has some beneficial, mostly weak, but potentially useful molecular effects, and it is basically free of major toxicity even at high clinical doses and on long-term treatment (Galasko *et al*, 2007). Its immunomodulatory effects, silencing of microglia and the regulatory effects on the endocannabinoid system prompted us to study the effects of R-flurbiprofen in experimental autoimmune encephalomyelitis (EAE) models of multiple sclerosis in mice. The primary hypothesis was that R-flurbiprofen may be useful in the treatment of MS-associated pain, because it reduces neuropathic pain in other models and because cannabis is known to be often the only effective medication for MS-associated pain. The results show that R-flurbiprofen indeed reduced EAE-evoked hyperalgesia in primary progressive PP-EAE in C57BL6/J mice and in relapsing-remitting RR-EAE in SJL mice. Most strikingly, however, R-flurbiprofen prevented or reduced the severity of EAE in both models in terms of clinical scores, spinal cord inflammation and cell invasion, gene regulation, encephalitis and optic neuritis and was associated with an increase in regulatory and inhibitory T cells and of the inhibitory cytokine IL-10.

## Results

### Prevention and cure of clinical EAE in C57BL6/J and SJL mice with R-flurbiprofen

**Figure 1:**
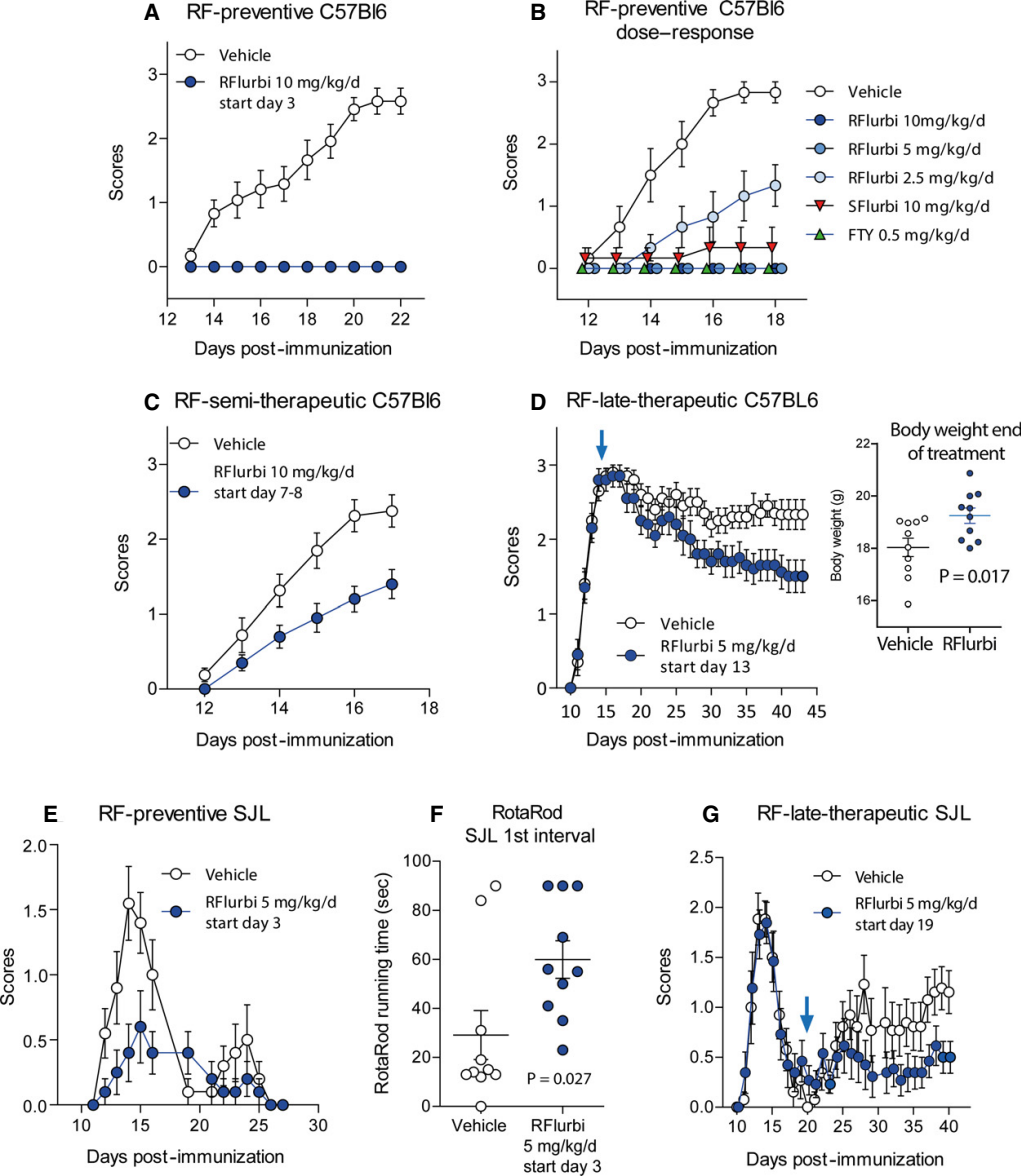
Attenuation of clinical EAE scores by R-flurbiprofen Time courses of the clinical scores (means ± SEM) in experimental autoimmune encephalomyelitis (EAE) models in C57BL6/J mice (A–D) and SJL/J mice (E–G) treated with vehicle versus R-flurbiprofen. C57BL6/J mice develop a primary progressive EAE (PP-EAE) and SJL/J a relapsing-remitting RR-EAE. Scores: Score 0, no obvious changes in motor functions; score 0.5, distal paralysis of the tail; score 1, complete tail paralysis; score 1.5, mild paresis of one or both hind legs; score 2, severe paresis of hind legs; score 2.5, complete paralysis of one hind leg; score 3, complete paralysis of both hind legs; score 3.5, complete paralysis of hind legs and paresis of one front leg.
EAE scores in C57BL6/J mice on preventive treatment with R-flurbiprofen (10 mg/kg/day) or vehicle. Treatment started 3 days after immunization and was continuously administered in the drinking water (*n* = 12 per group, rm-ANOVA for ‘treatment’ *P* = 3.691E-8).Dose response of EAE scores in C57BL6/J mice treated from 3 days after immunization with vehicle, R-flurbiprofen (2.5, 5, and 10 mg/kg/day), S-flurbiprofen (10 mg/kg/day) and FTY720 (0.5 mg/kg/day), all continuously administered in the drinking water (*n* = 6 per group, rm-ANOVA for ‘treatment’ with Dunnett *post hoc* versus vehicle, *P* < 0.0001 for all treatment groups and time points ≥ 14 days after immunization).EAE scores in C57BL6/J mice on semi-therapeutic treatment with R-flurbiprofen (10 mg/kg/day) or vehicle. Treatment started 3–4 days before onset of clinical scores, that is, 7–8 after immunization. (*n* = 10 per group, rm-ANOVA for ‘treatment’ *P* = 0.0085).EAE scores in C57BL6/J mice on late-therapeutic treatment with R-flurbiprofen (5 mg/kg/day) or vehicle. Treatment started after full development of the disease 13 days after immunization (the arrow indicates the start of treatment). Animals were allocated as score-matched pairs to the vehicle or R-flurbiprofen group. R-flurbiprofen or vehicle was administered via drug or vehicle soaked sweet cornflakes (*n* = 10 per group, rm-ANOVA for ‘time × treatment’ Greenhouse-Geisser adjusted, *P* = 0.008). The insert in (D) shows the mean body weight of the last 3 treatment days (*n* = 10 per group, two-sided unpaired Student's *t*-test, *P* = 0.017).EAE scores on preventive treatment with R-flurbiprofen (5 mg/kg/day) or vehicle. Treatment started 3 days after immunization and was continuously administered in the drinking water (*n* = 10 per group, rm-ANOVA for ‘treatment’, *P* = 0.0034).Running time in the Rota Rod test during the 1st interval in SJL mice treated with vehicle or R-flurbiprofen from day 3 onwards (5 mg/kg/day) (*n* = 10, unpaired two-sided Student's *t*-test, *P* = 0.027).EAE scores in SJL mice on late-therapeutic treatment with R-flurbiprofen (5 mg/kg/day) or vehicle. Treatment started on day 19, that is, after the first disease flare (arrow indicates the start). Animals were allocated to the treatment groups as score-matched pairs based on the severity of the first flare of the disease. Only mice with scores ≥ 1 were included, namely, 70% of all mice (*n* = 13 for the treatment period, *n* = 20 were immunized, rm-ANOVA for ‘time × treatment’, *P* = 0.0125).

Effects of the early and late onset of treatment with R-flurbiprofen were assessed in C57BL6/J mice (Fig 1A–D) that develop a non-remitting form of the disease, and in SJL mice (Fig 1E–G) that develop a relapsing-remitting (RR)-EAE. R-flurbiprofen completely prevented the development of clinical EAE scores in C57BL6/J mice when the treatment was started within 3 days after immunization (Fig 1A and B). This regimen is referred to as preventive treatment. The effect was dose-dependent (Fig 1B), and the minimum daily dose for complete prevention was 5 mg/kg/day. S-flurbiprofen, at 10 mg/kg/day, also prevented the development of EAE scores (Fig 1B). Effects of R-flurbiprofen were comparable to those of fingolimod (FTY720, 0.5 mg/kg/day), which was used as the positive control (Fig 1B). R-flurbiprofen also significantly reduced clinical EAE scores in C57BL6/J mice when treatment was started shortly before onset of clinical manifestations, referred to as semi-therapeutic (10 mg/kg/day, Fig 1C) and reduced clinical scores when the treatment was initiated after full development of the disease on day 13 (5 mg/g/day, Fig 1D). For the latter regimen, animals were allocated to R-flurbiprofen or vehicle as score-matched pairs according to their score on day 13.

R-flurbiprofen had similar efficacy in SJL mice with almost complete prevention of clinical scores in RR-EAE using preventive treatment (5 mg/kg/day Fig 1E). R-flurbiprofen significantly improved the Rota Rod running performance in these mice during the first remission of the disease, during which all mice had no or minor clinical scores (Fig 1F). In addition, R-flurbiprofen significantly reduced the EAE relapse rate and severity of RR-EAE scores when the treatment was started very late, 19 days after immunization, which was in the first remission (Fig 1G). The mice were allocated to the treatment groups as matched pairs, according to the severity of the first peak. This regimen is referred to as late-therapeutic. The results of statistical comparisons (rm-ANOVA or unpaired two-sided Student's *t*-tests), the number of animals and *P*-values are shown in Fig 1 in the respective graphs.

### Attenuation of nociceptive hypersensitivity in the EAE model

**Figure 2:**
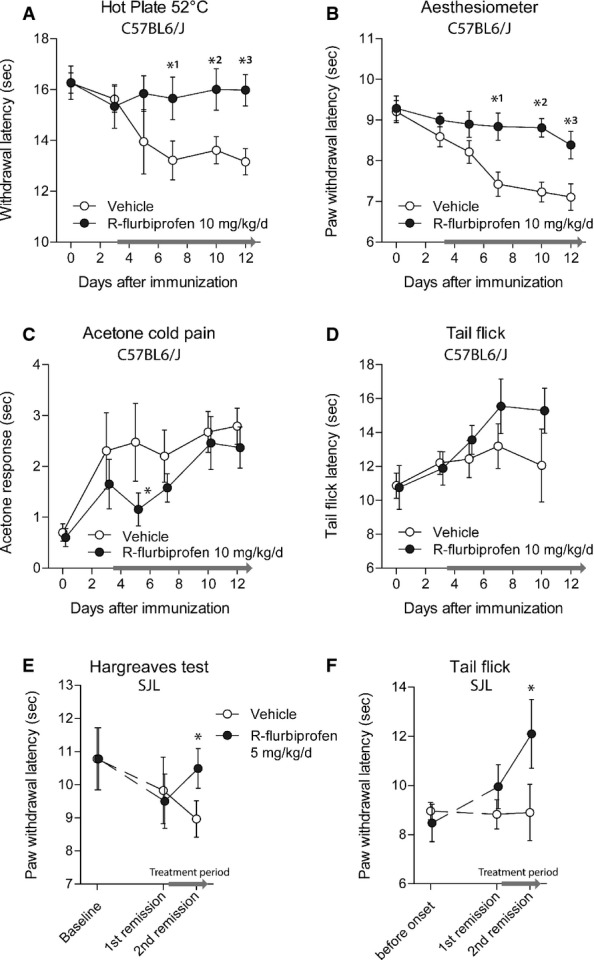
Reduction of EAE-evoked pain by R-flurbiprofen Time courses of the nociceptive behavior in the EAE model of multiple sclerosis in mice treated with vehicle or R-flurbiprofen, continuously administered via the drinking water. The treatment periods are indicated by a blue line on the *x*-axes. Data are the mean ± SEM. rm-ANOVAs revealed statistically significant differences between treatment groups, and asterisks indicate the time points, which differed significantly between groups.
Nociceptive sensitivity on heat stimulation in the hot plate test in C57BL6/J mice (*n* = 10, rm-ANOVA for ‘treatment’, *P* = 0.0107, *post hoc P*-values *^1^0.045, *^2^0.024, *^3^0.003).Nociceptive sensitivity on mechanical stimulation in the Dynamic Plantar test in C57BL6/J mice (*n* = 10, rm-ANOVA for ‘treatment’, *P* = 0.0008, *post hoc P*-values *^1^0.0034, *^2^0.0008, *^3^0.0142).Nociceptive sensitivity on cold stimulation in the acetone test in C57BL6/J mice (*n* = 10, rm-ANOVA, *post hoc* *P = 0.0354).Nociceptive sensitivity on heat stimulation in the tail flick test in C57BL6/J mice (*n* = 10).Nociceptive sensitivity on heat stimulation in the Hargreaves test in SJL mice (*n* = 6, unpaired two-sided Student's *t*-test for treatment period, *P = 0.048).Nociceptive sensitivity on heat stimulation in the tail flick test in SJL mice (*n* = 6, unpaired two-sided Student's *t*-test for treatment period, *P = 0.034).

R-flurbiprofen attenuates neuropathic pain in rodents after nerve injury (Bishay *et al*, 2010) and may therefore provide additional therapeutic benefit by reducing MS-associated pain. To test this hypothesis, nociception was analyzed in EAE mice before the onset of clinical scores and during remissions. In C57BL6/J mice, R-flurbiprofen administration (10 mg/kg/day, drinking water) was started 3 day after immunization. This treatment strongly reduced heat (Fig 2A) and mechanical (Fig 2B) hyperalgesia, which developed 4–5 days after immunization in vehicle treated mice. R-flurbiprofen also transiently reduced cold allodynia (Fig 2C). The tail flick assay did not reveal an EAE-evoked hypersensitivity but rather a subtle increase in the reflex time, which was not significantly altered by R-flurbiprofen (Fig 2D). In SJL mice, treatment with R-flurbiprofen and vehicle was started in score-matched pairs during the first remission of the disease. Under this late-treatment paradigm, R-flurbiprofen significantly reduced nociceptive hypersensitivity during the second remission (Fig 2E and F), that is, during the treatment period. The statistical comparisons, the number of animals and *P*-values are in the respective figure legend.

### Prevention of EAE-evoked immune cell activation and infiltration in the spinal cord

#### Immunofluorescence of myeloid cells in C57BL6/J mice

**Figure 3:**
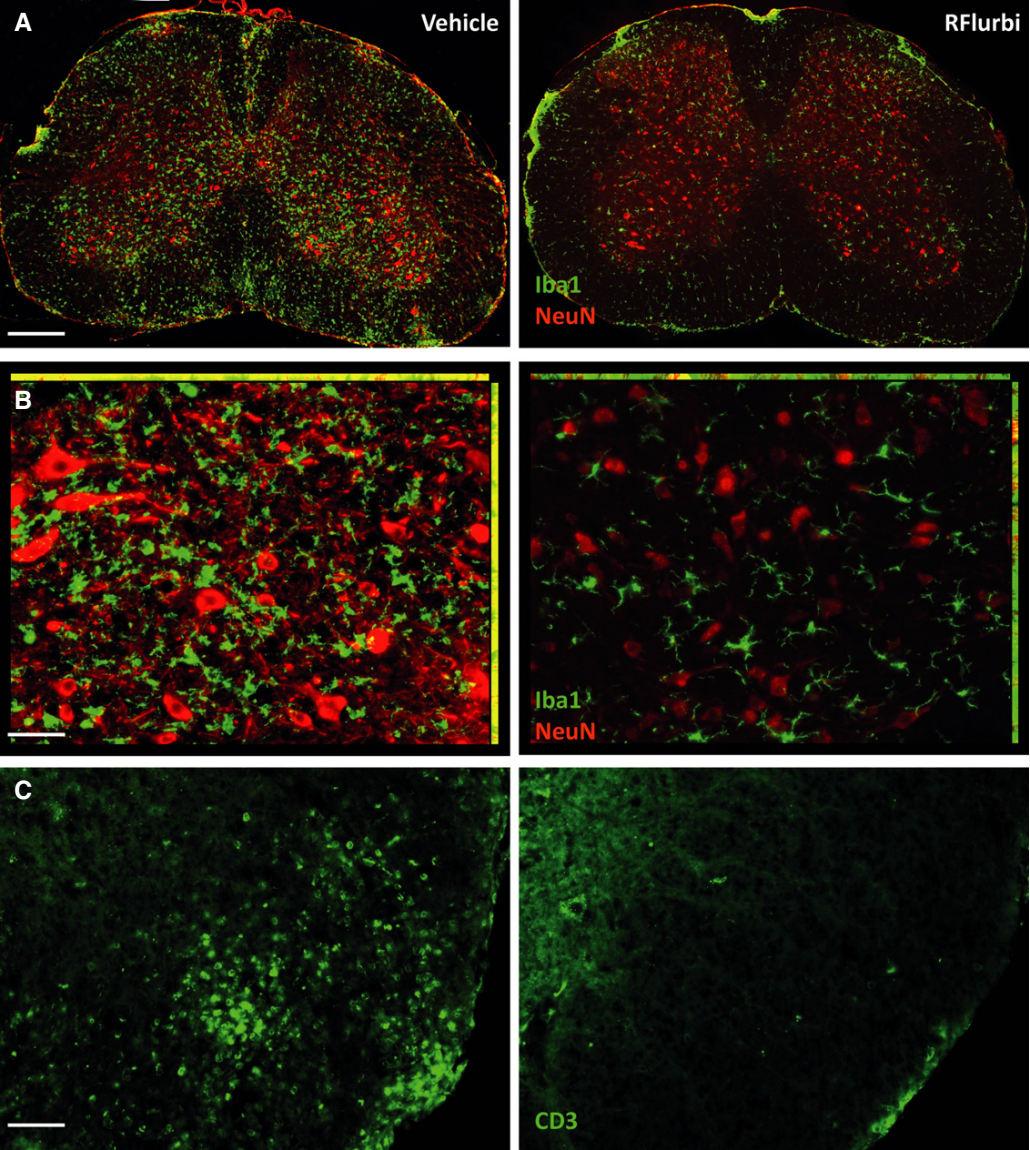
Immunofluorescence analysis of microglia, macrophages and T cells in the lumbar spinal cord in vehicle and R-flurbiprofen treated C57BL6/J mice (10 mg/kg/day) in the EAE model with preventive treatment (from day 3) The spinal cord was dissected out during the flare of the disease, day 22. Representative images of 4 mice per group.Immunofluorescence of ionized calcium binding adaptor molecule 1 (Iba-1, green) in microglia and macrophages. Neurons were counterstained with the neuronal marker NeuN in red. Scale bar 200 μm.Higher magnification of (A). Scale bar 50 μm.Immunofluorescence of the T-cell marker CD3 in the white matter of the lumbar spinal cord. Scale bars 50 μm.

Immune cell infiltration and resident microglia activation in the spinal cord were assessed per immunofluorescence and FACS analyses. Iba-1 immunofluorescence revealed a strong activation of resident microglia and infiltration of gray and white matter with monocytes/macrophages in vehicle treated C57BL6/J mice (Fig 3A and B left panel, antibodies listed in Supplementary Table S1), and CD3 immunofluorescence showed T-cell infiltrates (Fig 3C left). Myeloid and T-cell infiltrates were absent in R-flurbiprofen treated mice (Fig 3A–C right), and resident microglia mostly retained a branching ‘resting’ phenotype (Fig 3B right panel).

#### FACS analysis of the spinal cord in C57BL6/J mice

**Figure 4:**
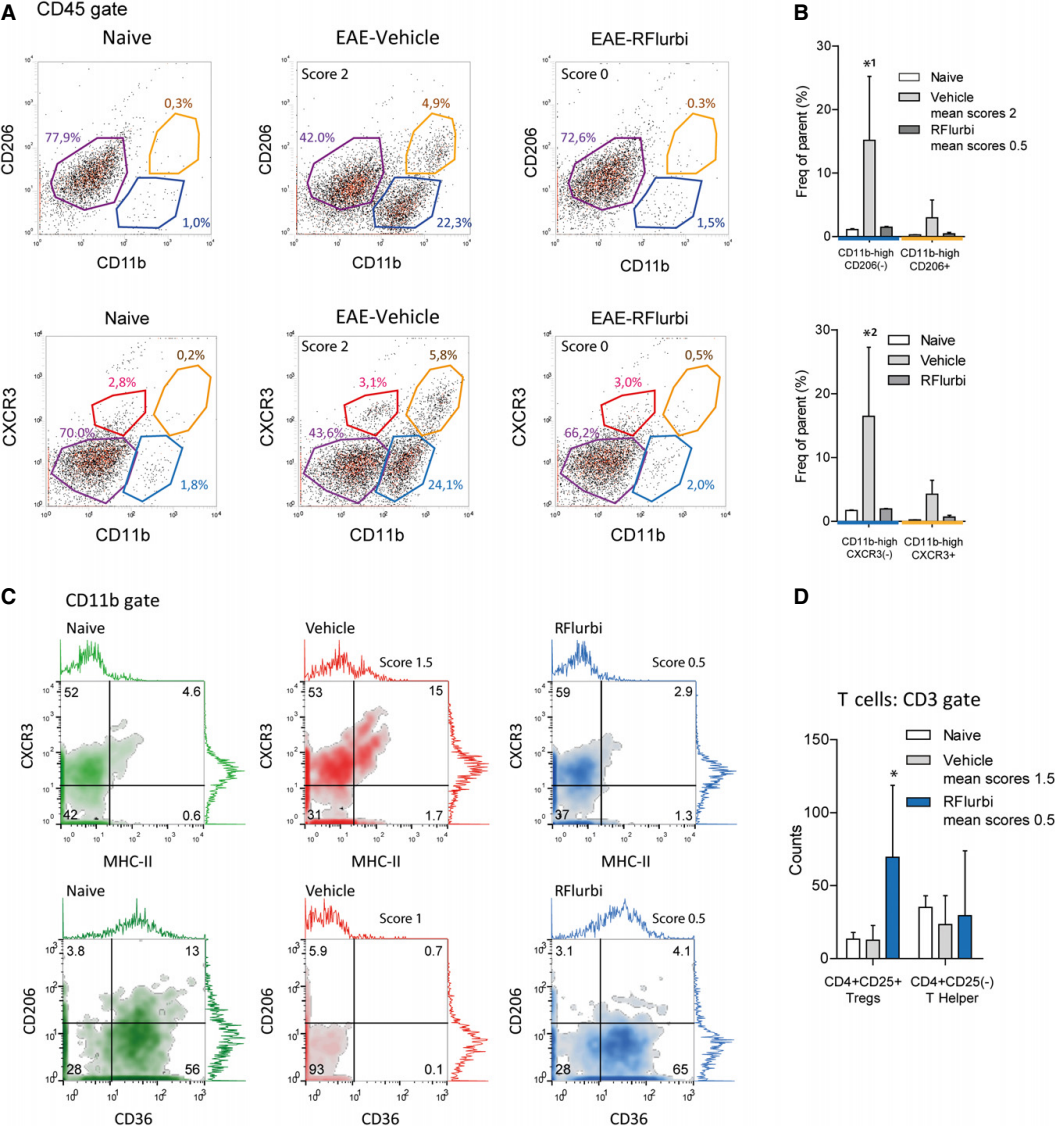
FACS analysis of CD11b and CD45 immunoreactive microglia/macrophages and CD3 positive T cells in the lumbar spinal cord of C57BL6/J naïve and EAE mice Further quantitative results in Supplementary Tables S2 and S3. The spinal cord was dissected out during the flare of the disease (day 20). The treatment with vehicle or R-flurbiprofen (10 mg/kg/day, drinking water) was preventive (A, B) or semi-therapeutic (C, D).Exemplary scatter dot plots of CD45^+^ cells, subsequently gated for CD11b versus CD206 (marker for M2-like microglia/macrophages) and CD11b versus CXCR3 (high in microglia). CD11b-high cells were increased in the vehicle group, but not in R-flurbiprofen treated mice.Quantitative results of the blue and yellow gates of (A). (*n* = 3, two-way ANOVA, *post hoc* Bonferroni versus naïve for ‘treatment’ *P*-values *^1^0.0093, *^2^0.005).Exemplary density plots of CD11b^+^ cells subsequently gated for MHC-II (marker for antigen presenting cells) versus CXCR3 and CD36 (scavenger receptor class B, marker for M2-like) versus CD206. CD36^+^ M2-like cells were strongly reduced in the vehicle group.Quantitative result for CD3^+^ T cells subsequently gated for CD4 versus CD25. CD4^+^CD25^+^ T cells (likely to be Tregs) were higher in the R-flurbiprofen group (*n* = 5, two-way ANOVA, *post hoc* Dunnett versus naïve for ‘treatment’ *P = 0.0056).

Morphologic results of the spinal cord were quantitatively confirmed per FACS analyses (Fig 4, Supplementary Table S2). The EAE-evoked increase in CD11b^+^ and CXCR3^+^ myeloid cells did not occur in R-flurbiprofen treated mice (Fig 4A and B). The cell populations in the spinal cord in R-flurbiprofen treated EAE mice were similar to those of naïve mice. When the treatment with R-flurbiprofen was started later, 4 days before onset of the clinical scores (Fig 4C and D), the overall number of CD45^+^ cells did not significantly differ between treatment groups but CD11b^+^ myeloid cells were reduced (Supplementary Table S3). In addition, gating for CD11b subpopulations revealed a decrease in MHC-II^+^ antigen presenting cells and an increase in CD36^+^ (scavenger receptor class B) M2-like myeloid cells in response to R-flurbiprofen (Fig 4C, Supplementary Table S3). Analyses of CD3^+^ T cells in the spinal cord showed a relative increase of CD4^+^CD25^+^ T cells in R-flurbiprofen treated mice, which are likely to be regulatory T cells (Fig 4D). FACS antibodies are listed in Supplementary Table S1.

#### Spinal cord immune cell invasion: BMX from β-actin-EGFP donor mice

**Figure 5:**
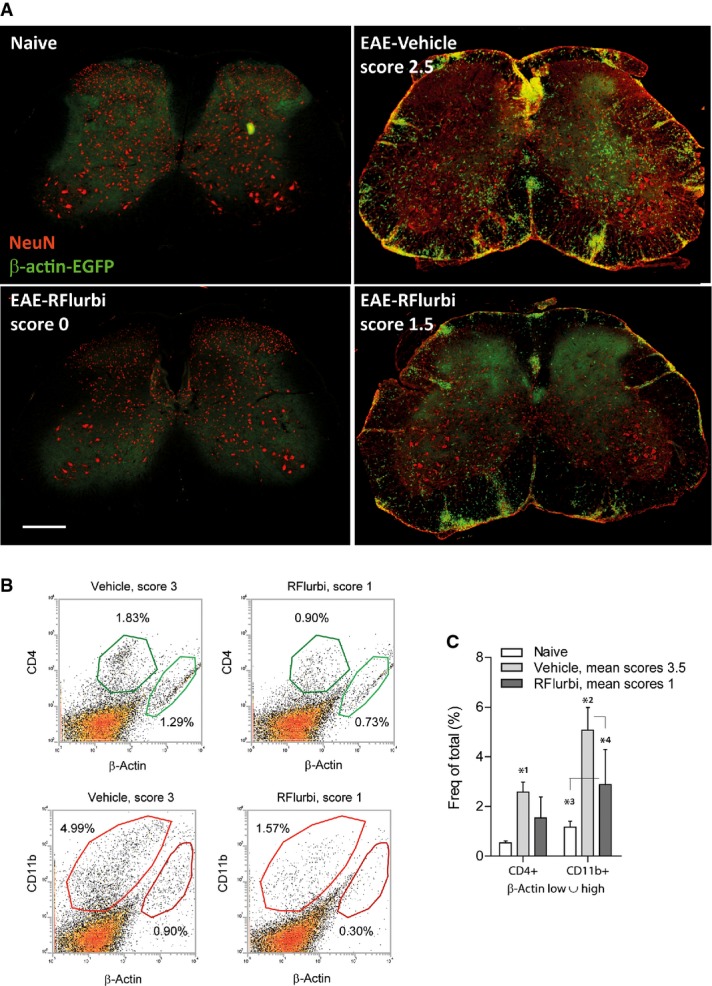
Analysis of immune cell infiltration into the spinal cord in naïve and EAE mice after transplantation of bone marrow (BMX) from β-actin-EGFP donor mice BMX was performed 3 weeks before immunization. Mice (C57BL6/J) were treated with vehicle or R-flurbiprofen (10 mg/kg/day, drinking water) from the 3rd day after immunization, and tissue was dissected out during the flare of the disease (day 16). BMX treated mice generally develop stronger and earlier EAE.Immunofluorescence of β-actin-EGFP positive infiltrating cells (green). Neurons were counterstained with the neuronal marker NeuN (red). Scale bars 200 μm. Two extreme examples of the R-flurbiprofen treated group with complete and moderate efficacy are shown.Scatter dot plots of the FACS analysis of β-actin-EGFP positive infiltrating T cells (CD4^+^) and myeloid cells (CD11b^+^).Quantitative results of the ‘β-actin low or high’ gate. Myeloid cell infiltrates were significantly reduced in the R-flurbiprofen group. Further quantitative results are given in Supplementary Table S4. The asterisks indicate statistically significant differences versus naïve and as indicated (two-way ANOVA, *post hoc* Bonferroni for ‘treatment’, *n* = 5 per group, *P*-values *^1^0.0113, *^2^0.000962, *^3^0.0422, *^4^0.0059).

To assess the relative contribution of invading immune cells and resident microglia, we used a bone marrow transplant model with β-actin-EGFP mice as donors. EAE was induced after consolidation, and R-flurbiprofen or vehicle treatments started 3 days after immunization. Immunofluorescence and FACS analyses of β-actin positive cells showed a strong reduction of cell invasion into the spinal cord. Two examples of spinal cords of R-flurbiprofen treated mice in Fig 5A show the extremes of R-flurbiprofen effects, that is, complete prevention up to moderate reduction. FACS analyses (Fig 5B,C; Supplementary Table S4) quantitatively confirmed the morphologic results and showed a reduction of both CD11b^+^ myeloid cells and CD4^+^ T-helper cells.

#### FACS analysis of splenocytes: increase in Tregs and IL-10

**Figure 6:**
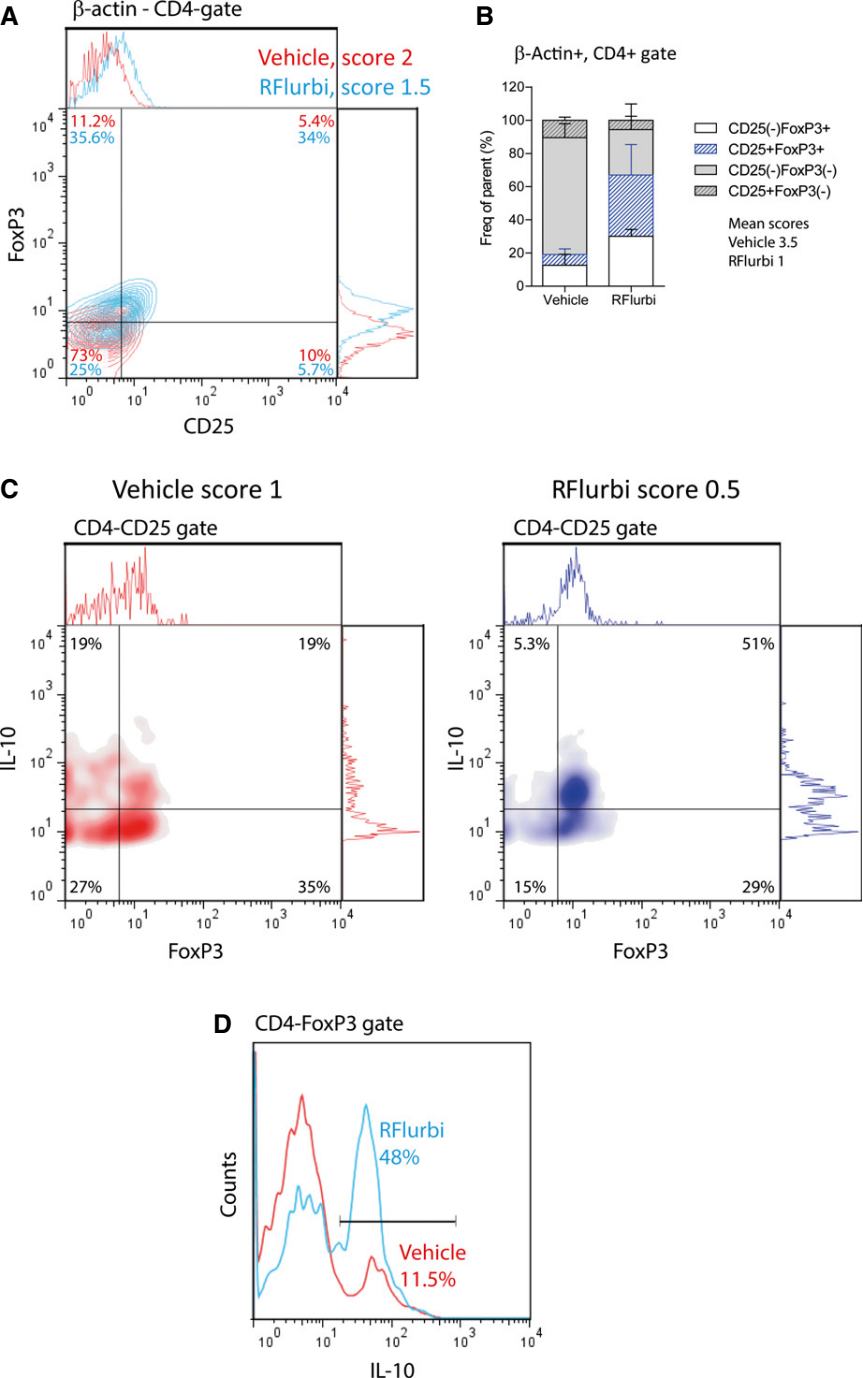
FACS analyses of splenocytes during EAE of bone marrow transplanted C57BL6/J mice treated with vehicle or R-flurbiprofen (10 mg/kg/day, drinking water) The BMX from β-actin-EGFP mice was done 3 weeks before immunization. Treatment started 3 days after immunization, and spleens were dissected out during the flare of the disease, day 16. Score-matched pairs were analyzed to assess effects on T-cell subpopulations. Infiltrating T-helper cells were identified as being β-actin^+^CD4^+^ and were subsequently gated for CD25 versus FoxP3 to identify Tregs (CD25^+^FoxP3^+^).Quantitative analysis of CD4^+^CD25^+^FoxP3 Tregs (blue bars). (*n* = 4 mice, two-way ANOVA ‘population × treatment’ Greenhouse-Geisser *P* = 0.003. Further quantitative results are given in Supplementary Table S5.Density plots of CD4^+^CD25^+^ splenocytes from C57Bl6/J mice with EAE and semi-therapeutic treatment with vehicle or R-flurbiprofen (10 mg/kg/day, drinking water). Spleens were dissected out during the flare, day 18. CD4^+^CD25^+^ were subsequently gated for IL-10 versus FoxP3.IL-10 histogram of CD4^+^FoxP3^+^ splenocytes from the same mice. Quantitative results are given in Supplementary Table S6.

FACS analyses of splenocytes from bone marrow transplanted C57BL6/J mice with EAE showed that R-flurbiprofen treatment increased the frequency of CD25^+^FoxP3^+^ regulatory CD4^+^ T cells (Fig 6A,B; Supplementary Table S5). Further FACS analyses of splenocytes prepared from C57BL6/J mice treated with a semi-therapeutic regimen (start 4 days before onset) confirmed the increase in CD4^+^CD25^+^FoxP3^+^ Tregs in the R-flurbiprofen group (Supplementary Table S6) and revealed a strong increase in the inhibitory cytokine IL-10 (Fig 6C,D, Supplementary Table S6).

### Attenuation of immune cell infiltration and myelin destruction in RR-EAE in SJL mice

**Figure 7:**
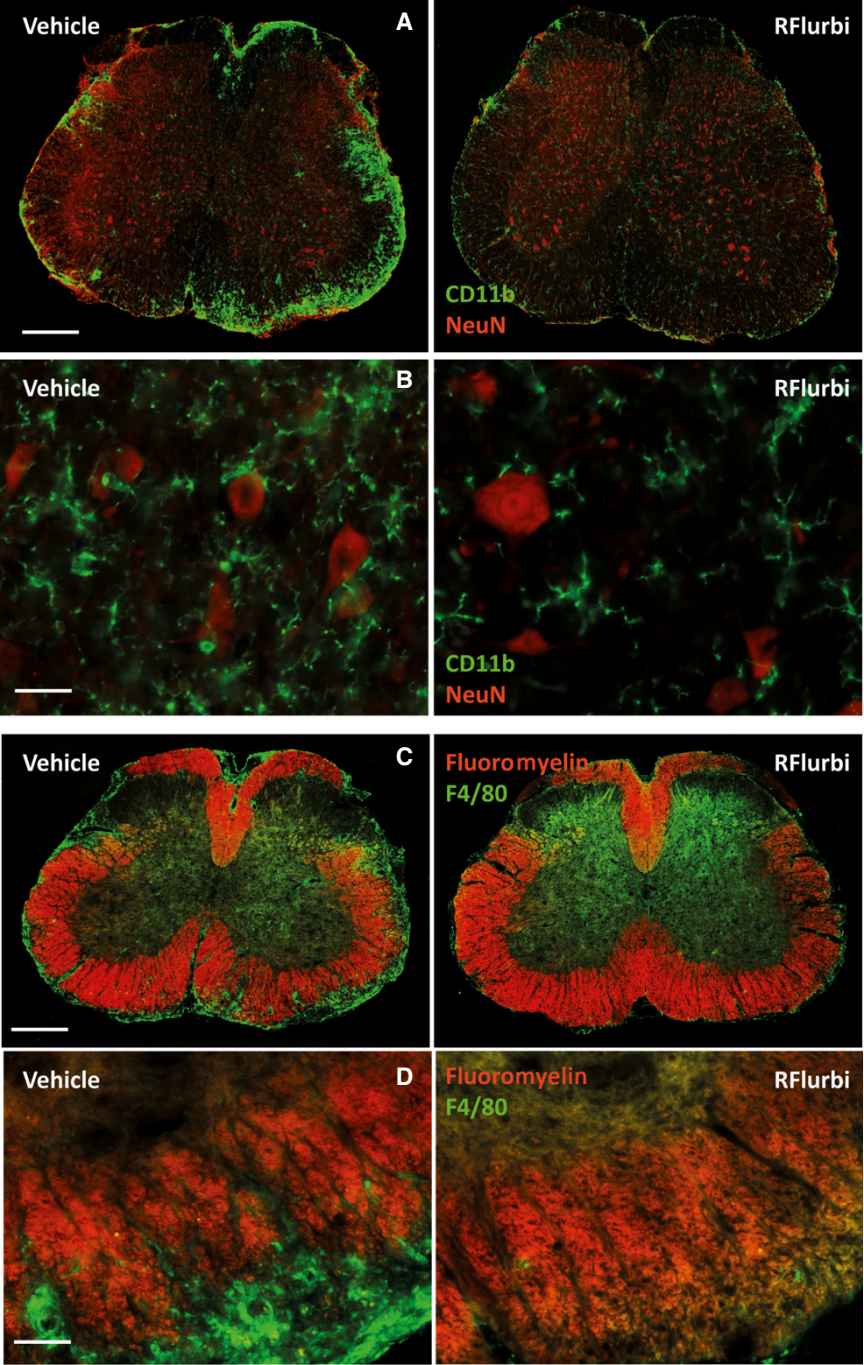
Reduction of EAE-evoked immune cell infiltration and demyelination in SJL mice by R-flurbiprofen Immunofluorescence analysis of the inflammation and demyelination in the lumbar spinal cord in the EAE model of multiple sclerosis in SJL mice, which received late-therapeutic treatment with R-flurbiprofen or vehicle (5 mg/kg/day, start of treatment day 19, time of dissection day 42). Representative images of 4 mice per group.CD11b immunoreactive macrophages and microglia (green) in vehicle and R-flurbiprofen treated mice. Neurons were counterstained with the neuronal marker NeuN (red). Scale bar 200 μm.Higher magnification of A of the gray matter of the ventral horn. Scale bar 50 μm.Immunofluorescence of myelin (fluoromyelin, red) and macrophages (F4/80, green) in vehicle and R-flurbiprofen treated mice. Scale bar 200 μm.Higher magnification of C of the ventral horn. Scale bar 50 μm.

The therapeutic efficacy of R-flurbiprofen for relapsing-remitting EAE was tested in SJL mice with a late-therapeutic regimen, that is, all mice were left untreated during the first flare of the disease up to the first remission and were then pairwise allocated to the treatment groups based on their initial maximum score. Only mice with scores > 1 during the first flare were included. Spinal cords and splenocytes were prepared during the 3rd remission. The mice had clinical scores ≤ 0.5 at this time. In vehicle treated mice, CD11b and F4/80 immunofluorescence showed strong infiltrates of monocytes/macrophages, mainly in the white matter (Fig 7A,C and D left panel), accompanied by microglial proliferation and activation in the gray matter (Fig 7B left), the latter indicated by the phagocyte-like rhomboid phenotype. Microglia did not show an activated phenotype in R-flurbiprofen treated groups (Fig 7B right panel), and infiltrates around myelinated fibers were almost completely absent. Vehicle treated mice had patches of destroyed myelin (Fig 7C and D left), mainly in ventral regions, which did not occur in R-flurbiprofen treated mice (Fig 7C and D right).

### Increase of CTLA-4 positive inhibitory T cells in RR-EAE in SJL mice

**Figure 8:**
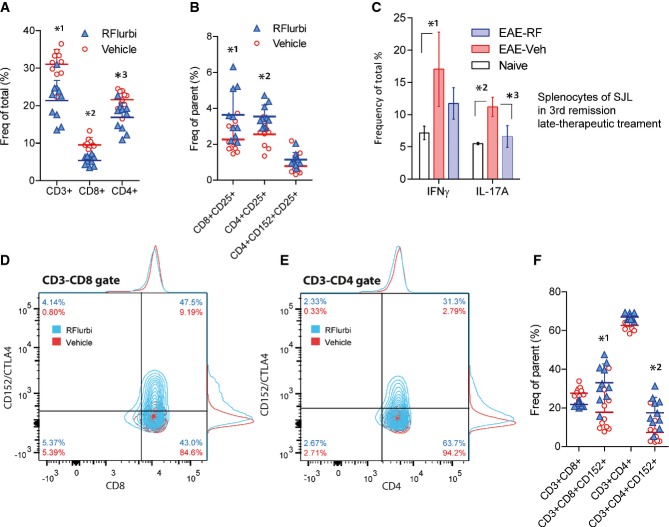
FACS analysis of splenic T-cell subpopulations and intracellular cytokines in SJL EAE mice treated with vehicle or R-flurbiprofen (5 mg/kg/day, drinking water, *n* = 10 per group) Splenocytes were obtained from SJL mice with EAE during the 1st flare of the disease (A, B, D–F; start of treatment day 5, dissection day 15) or during the 3rd remission (C; start of treatment day 19, dissection day 43).Scatter dot plot of CD3^+^, CD4^+^ and CD8^+^ T cells (two-way ANOVA for ‘treatment’ *P* = 0.0003, *post hoc* for each population *P*-values *^1^0.0015, *^2^0.0281, *^3^0.0104).Scatter dot plot of CD25^+^ cells. R-flurbiprofen significantly increased the frequency of CD8^+^CD25^+^ and of CD4^+^CD25^+^ regulatory T cells (two-way ANOVA for ‘treatment’ *P* = 0.0047, *post hoc* for each population *P*-values *^1^0.0013, *^2^0.0267).Intracellular cytokines of *n* = 3 mice per treatment group after *ex vivo* restimulation with PMA/ionomycin. IFNγ and IL17A positive cells were reduced in the R-flurbiprofen group (two-way ANOVA for ‘treatment’ *P* = 0.0102, *post hoc* for each cytokine *P*-values *^1^0.0009, *^2^0.0262, *^3^0.0359).Exemplary contour plots of CD8^+^CD152/CTLA4^+^ cells. CD152/CTLA4 is a marker for T cells that down-regulate an activated immune system.Exemplary contour plots of CD4^+^CD152/CTLA4^+^ cells.Quantitative results for CD152/CTLA4 positive cells. R-flurbiprofen significantly increased CD3^+^CD8^+^CD152^+^ and CD3^+^CD4^+^CD152^+^ cells [two-way ANOVA (population and treatment) for ‘treatment’ *P* = 0.0035, *post hoc* for each population *P*-values *^1^0.0003, *^2^0.0033, further quantitative results in Supplementary Table S7].

T-cell subpopulations in the spleen were studied in SJL mice during the first flare of the disease, on day 15 after immunization. R-flurbiprofen was started 5 days after immunization to allow for some immune activation. The number of CD4^+^ or CD8^+^ T cells was significantly lower in the R-flurbiprofen treatment group (Fig 8A), but in both T-cell subpopulations R-flurbiprofen treated mice had increased fractions of CD25^+^ T cells, which are likely to be regulatory T cells (Fig 8B, Supplementary Table S7). Analysis of intracellular cytokines revealed high frequencies of IFNγ and IL-17A positive cells in vehicle treated mice, whereas levels in R-flurbiprofen treated mice were similar to those of naïve controls (Fig 8C). In the CD3^+^ gate, CD4^+^ or CD8^+^ T cells were further gated for CD152/CTLA-4, which is a marker for T cells, that down-regulate the immune system. R-flurbiprofen treatment caused a strong increase in the relative numbers of CD152/CTLA-4 positive T cells (Fig 8D–F, Supplementary Table S7), both in CD4^+^ and CD8^+^ populations.

### *In vivo* imaging of optic neuritis and brain inflammation in SJL mice

**Figure 9:**
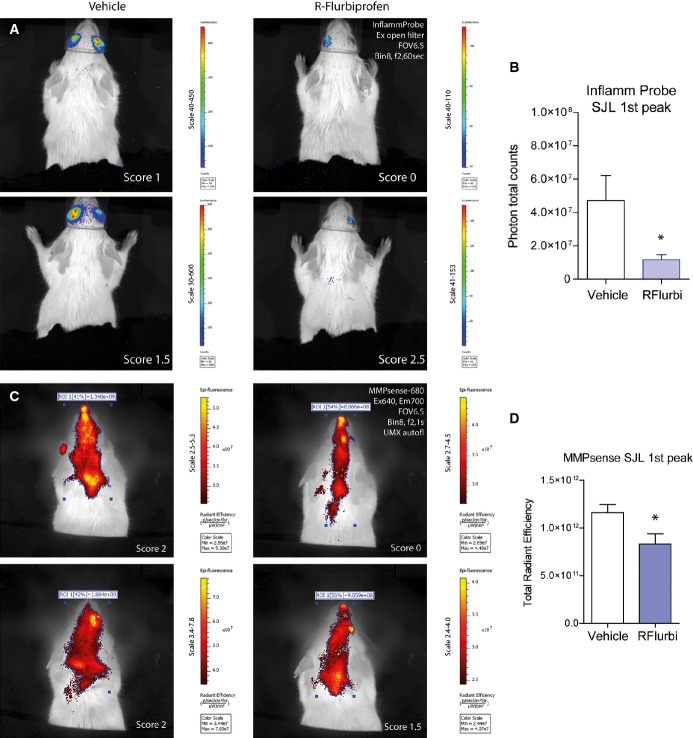
*In vivo* imaging of optic neuritis and brain inflammation in the EAE model of multiple sclerosis in SJL mice Images were captured during the 1st flare of the disease, day 13 in mice treated with vehicle or R-flurbiprofen (5 mg/kg/day).Bioluminescent images of optic neuritis captured 10 min after i.p. injection of the Inflammation Probe (2 examples of *n* = 8). Treatment started 5 days after immunization.Quantification of total photon counts of the Inflammation Probe in regions of interest (unpaired two-sided Student's *t*-test, *P = 0.0255).Epi-fluorescent unmixed images of brain and spinal cord inflammation captured 24 h after intravenous injection of the near-infrared MMPsense-680 probe (2 examples of *n* = 8). Treatment started 3 days after immunization.Quantification of total radiant efficiency of MMPsense after spectral unmixing of autofluorescence (unpaired two-sided Student's *t*-test, *P = 0.0273).

Bioluminescence and near-infrared *in vivo* imaging were used to assess the brain inflammation and optic neuritis *in vivo*. SJL mice were imaged during the first flare of the disease (Inflammation probe, Fig 9A and B; MMPsense, Fig 9C and D) to observe the inflammation of the optic nerve and brain. The Inflammation Probe, a bioluminescent marker activated by peroxidases, mainly detected optic neuritis, which was significantly stronger in vehicle than in R-flurbiprofen treated mice (Fig 9B). MMPsense, which is a near-infrared probe activated by metalloproteinases, revealed the inflammation both in the brain and in the spinal cord, which was significantly reduced in the R-flurbiprofen treatment group (Fig 9D).

### Blood-brain barrer leakage and myelin destruction

Near-infrared *in vivo* imaging was used to assess the leakage of the blood-brain barrer (Fig 10A and B; SJL mice) and myelin inflammation (Fig 10C and D; C57BL6 mice). A late-treatment strategy was used in these experiments, and imaging was performed at the end of the observation period, that is, SJL mice treated with R-flurbiprofen or vehicle from day 19 on were imaged during the 3rd remission to assess the disruption of the blood-brain barrer (BSA-Cy5.5, Fig 10A and B), and C57BL6 mice treated with R-flurbiprofen or vehicle from day 13 were imaged on day 39 to assess myelin inflammation (DBT; Fig 10C and D).

**Figure:**
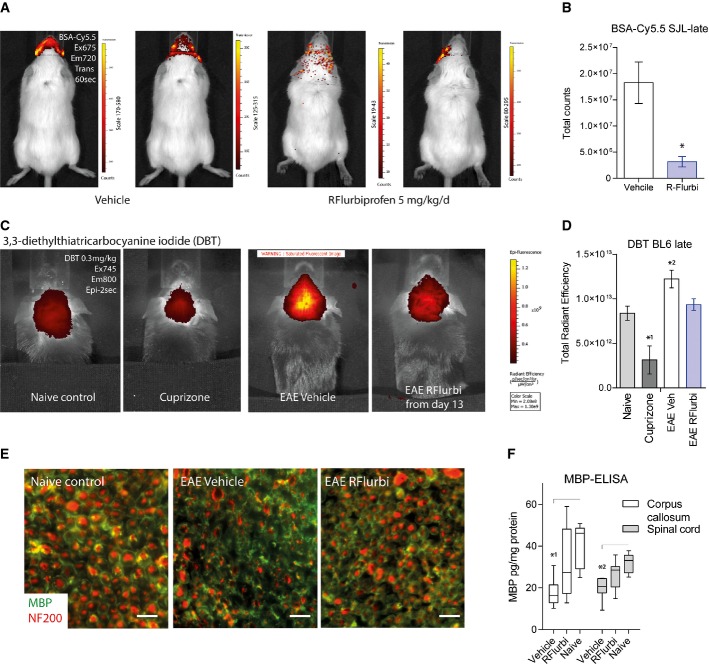
Blood-brain barrer disruption and myelin inflammation and destruction in the EAE model of multiple sclerosis in SJL and C57BL6/J mice *In vivo* imaging of blood-brain barrer disruption 4–6 h after intravenous injection of Cy5.5-labeled BSA in SJL mice treated with vehicle or R-flurbiprofen (5 mg/kg/day) from day 19 after immunization onwards. Mice were imaged during the 3rd remission. Cy5.5-labeled BSA accumulates in the brain only if the permeability of the BBB is pathologically increased.Quantitative results for the total fluorescent counts of BSA-Cy5.5 in regions of interest (*n* = 2 mice, 3 time points each, unpaired two-sided Student's *t*-test, *P = 0.0006).Representative *in vivo* near-infrared fluorescence (NIRF) images of the myelin-binding dye, DBT in the brain of a naive C57BL6 mouse, a mouse treated with the demyelination agent, cuprizone for 6 weeks and in brains of EAE mice treated from day 13 after immunization with vehicle or R-flurbiprofen (5 mg/kg/day) for 26 days.Quantitative results for the total radiant efficiency of DBT NIRF signals in the region of interest (*n* = 4 mice per group). Univariate ANOVA with Bonferroni *post hoc* analysis versus naive *^1^0.0213, *^2^0.0136.Immunofluorescence analysis of the optic nerve in naïve mice and in EAE mice (C57BL6/J) treated with vehicle or R-flurbiprofen (10 mg/kg/day, drinking water) from the 5th day after immunization. Myelin was immunostained with anti-myelin basic protein (MBP, green), and axons were counterstained with anti-neurofilament 200 (NF200, red). Representative images of *n* = 3 mice per group. Scale bars are 20 μm.Quantitative analysis of myelin basic protein (MBP) levels in the corpus callosum and spinal cord using an enzyme immune assay in naïve mice and in C57BL6/J EAE mice treated from day 13 after immunization with vehicle or R-flurbiprofen (5 mg/kg/day) for 28 days (*n* = 7 per group). Two-way ANOVA (‘region’ and ‘treatment’) *P* = 0.0002 for ‘treatment’, *post hoc* analysis *^1^0.0022, *^2^0.0089).

BSA labeled with Cy5.5 normally remains in the vasculature unless there is a leakage. A disruption of the blood-brain barrer causes its accumulation in the brain. Visualization of the brain accumulation by near-infrared imaging revealed a much stronger disruption of the BBB, mainly around the eyes, in the vehicle group as compared to R-flurbiprofen treated mice (Fig 10A and B).

The process of myelin destruction was imaged with the near-infrared dye DBT that binds to myelin (Fig 10C and D). Its binding was reduced in the cuprizone-evoked demyelination model as described (Wang *et al*, 2011) (control group) but significantly increased in EAE mice treated with vehicle whereas the DBT fluorescence was almost normal in R-flurbiprofen treated mice.

Myelin inflammation was also assessed by immunofluorescence of myelin basic protein, MBP in the optic nerve in C57BL6/J mice during the flare of EAE and semi-therapeutic treatment with R-flurbiprofen (Fig 10E). The myelin structure was strongly destroyed in the vehicle group but largely intact in the R-flurbiprofen group. Neurofilament 200 staining additionally showed destruction of the axons in the vehicle group, whereas the axon morphology in the R-flurbiprofen group was similar to that in the naïve controls.

For further analysis of myelin destruction, myelin basic protein (MBP) was quantified with an enzyme immunoassay in the corpus callosum and spinal cord in C57BL6/J mice with a late onset of R-flurbiprofen treatment (Fig 10F). The tissue was excised 41 days after immunization (28 days of treatment). MBP levels were strongly reduced in vehicle treated mice but much less in R-flurbiprofen treated mice. The R-flurbiprofen group did not significantly differ from the naïve control mice (*P*-values and *n* numbers in the legend of Fig 10).

### Reduction of EAE-evoked upregulation of pro-inflammatory genes in the spinal cord

**Figure 11:**
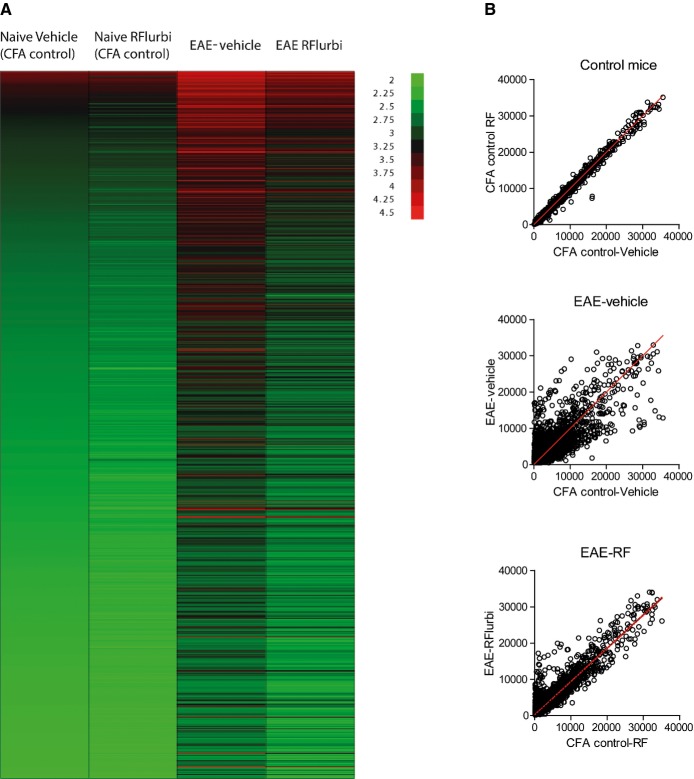
Reduction of EAE-evoked upregulation of pro-inflammatory genes in the spinal cord by R-flurbiprofen Microarray mRNA analysis of the lumbar spinal cord in EAE and sham control mice treated with vehicle or R-flurbiprofen (10 mg/kg/day, drinking water) from the 5th day after immunization. Spinal cords were dissected out during the flare, day 16. Sham control mice were injected with CFA without MOG35-55. Three samples were analyzed per group, and each sample was comprised of pooled tissue from 3 mice, that is, the array data are based on *n* = 9 mice per group. Top 100 regulated genes in Supplementary Table S8.Heat map of log transformed mRNA expression of genes, which were upregulated ≥ twofold in the EAE-vehicle group (green low intensity, red high intensity).Scatter plots of mRNA intensities. CFA control mice (no EAE) treated with vehicle or R-flurbiprofen (top), vehicle treated CFA control mice versus vehicle treated EAE mice (middle) and R-flurbiprofen treated CFA control mice versus R-flurbiprofen treated EAE mice (bottom).

Microarray gene expression analysis of the lumbar spinal cord in sham and EAE mice showed the expression of *n* = 5438 genes. A number of 563 genes were down-regulated by at least 50% in the EAE-vehicle group. Only 20 genes fulfilled these criteria in the R-flurbiprofen group, and R-flurbiprofen had no effect on gene expression in sham groups, that is, those injected with CFA without MOG (Fig 11A and B). EAE caused a > twofold upregulation of 1022 genes in the vehicle group and of 529 genes in the R-flurbiprofen treated group (color plots of upregulated genes in Fig 11A). A number of 523 genes were less upregulated (> 1.5-fold difference) in the R-flurbiprofen group (top 100 listed in Supplementary Table S8). The biological roles of these 523 differentially upregulated genes were identified by means of over-representation analysis (ORA) (Backes *et al*, 2007) using the Web-based GeneTrail tool (http://genetrail.bioinf.uni-sb.de/) (Keller *et al*, 2008) (Supplementary Table S9). This compared terms annotated to the differentially upregulated genes in the Gene Ontology database (GO; http://www.geneontology.org/) (Ashburner *et al*, 2000) with the occurrence of terms among genes expressed in the spinal cord (ORA parameters: *P*-value threshold *t*_*p*_ = 0.05 and Bonferroni α correction). The resulting knowledge representation graph showed that the effects of R-flurbiprofen mainly involved genes coding for proteins located in the extracellular region (GO:0005576, expected: 34 genes, found: 106 genes, *P* = 1.3 × 10^−26^) whereas genes with intracellularly located products were significantly underrepresented (GO:0005622, expected: 346 genes, found: 286 genes, *P* = 4.3 × 10^−7^). A second result was that the molecular function of the upregulated gene products converged toward transmembrane receptor activity (GO:0004888, *P* = 6.6 × 10^−11^). To reduce the complexity of the GO term hierarchy, functional areas were derived consisting of headline terms that represented the gene set at a maximum of certainty, information value, coverage and conciseness (Lotsch *et al*, 2013). This analysis revealed that R-flurbiprofen had consequences for genes involved in the response to stimulus (GO:0050896, *P* = 2.2 × 10^−14^) including inflammatory responses (GO:0006954, *P* = 2.0 × 10^−12^). Further main processes less upregulated in the R-flurbiprofen treated group were immune system processes (GO:0002376, *P* = 2.4 × 10^−10^), signal transduction (GO:0007165, *P* < 0.001), signaling pathway (GO:0023033, *P* = 6.0 × 10^−5^), cell motility (GO:0048870, *P* < 0.001), cell proliferation (GO:0008283, *P* < 0.001) and biological adhesion (GO:0022610, *P* = 9.3 × 10^−8^). By contrast, genes involved in metabolic processes, in particular those relevant for gene expression (GO:0010467, *P* < 0.001), were significantly underrepresented among biological processes affected by R-flurbiprofen administration (Supplementary Table S9). The graphical representation of the gene ontology (GO) classification showing the polyhierarchy of functional annotations (GO terms) assigned to those genes that were less upregulated in EAE mice treated with R-flurbiprofen as compared to vehicle treated is shown in Supplementary Fig S1.

### Pharmacokinetics, prostaglandins and endocannabinoids

**Figure 12:**
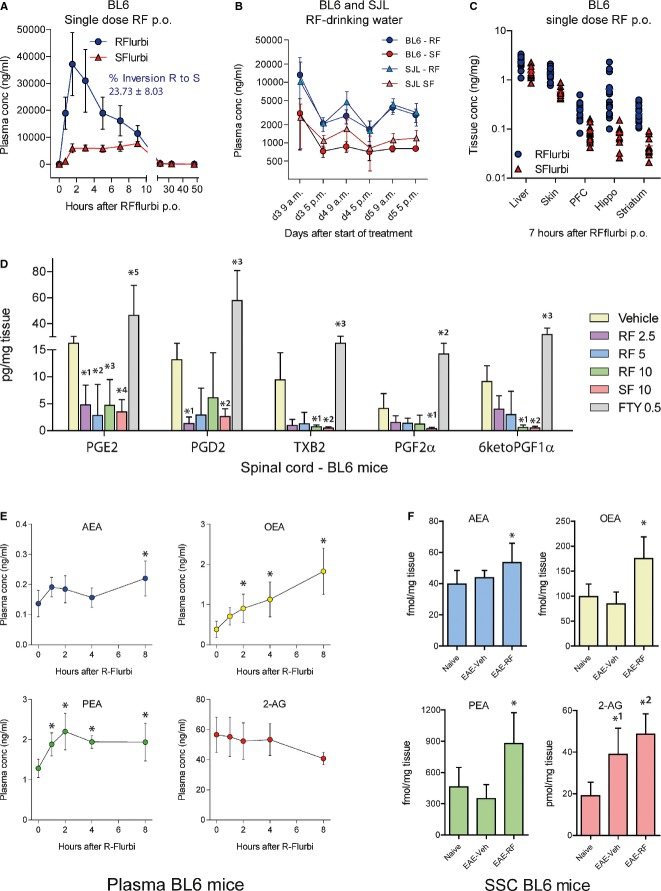
Plasma and tissue concentrations of R-flurbiprofen in C57BL6/J and SJL mice and pharmacodynamic effects in terms of prostaglandins and endocannabinoids Plasma concentration versus time courses in C57BL6/J mice (*n* = 14) after a single oral dose of 10 mg/kg R-flurbiprofen. The rates of R to S inversion in (A) and (B) were calculated on the basis of the AUCs up to 8 h.Comparison of the circadian rhythms of R- and S-flurbiprofen plasma concentrations in C57BL6/J and SJL mice receiving continuous R-flurbiprofen (10 mg/kg/day) in the drinking water (*n* = 5 per group).Tissue concentrations of R-and S-flurbiprofen in C57BL6/J mice in liver, skin and three brain regions (PFC, prefrontal cortex; Hippo, hippocampus) 7 h after a single oral dose of 10 mg/kg R-flurbiprofen (*n* = 14).Prostaglandin concentrations in the spinal cord of C57BL6/J mice in the EAE model. The mice received preventive treatment with vehicle, R-flurbiprofen (2.5, 5, 10 mg/kg/day), S-flurbiprofen (10 mg/kg/day) or FTY (0.5 mg/kg/day) from the 3rd day after immunization, and the tissue was dissected out during the flare of the disease, day 20. (*n* = 6 per group, two-way ANOVA (‘lipid’ and ‘treatment’) with subsequent Dunnett *post hoc* analyses versus vehicle. *P*-values of *post hoc* tests PGE2: *^1^0.0014, *^2^0.0002, *^3^0.0013, *^4^0.0004, *^5^0.0001; PGD2: *^1^0.0275, *^2^0.050, *^3^0.0001; TXB2: *^1^0.0405, *^2^0.0351, *^3^0.0024; PGF2α: *^1^0.0395, *^2^ 0.0172; 6ketoPGF1α: *^1^0.0440, *^2^0.0428, *^3^0.0001).Endocannabinoid concentrations in plasma of C57BL6/J mice before and after receiving a single i.p. injection of 10 mg/kg R-flurbiprofen during the flare of EAE (*n* = 5). rm-ANOVA within subject factor ‘time’ for AEA *P* = 0.0061, OEA *P* = 0.0001, PEA *P* = 0.0010, 2-AG 0.0016. The asterisks indicate those time points that differed significantly versus baseline.Endocannabinoid concentrations in the somatosensory cortex (SSC) of naïve and EAE mice (C57BL6/J) treated continuously with vehicle or R-flurbiprofen (10 mg/kg/day in drinking water) from the 3rd day after immunization (*n* = 13 per group). The tissue was dissected out during the flare of the disease, day 20 (univariate ANOVA with *post hoc* Bonferroni for AEA: *P = 0.020; OEA: *P = 0.000052; PEA: *P = 0.000412; 2-AG: *^1^*P* =0.0002, *^2^*P* = 0.00003). Data information: Abbreviations in (E) and (F): AEA, anandamide; OEA, oleoylethanolamide; PEA, palmitoylethanolamide; 2-AG arachidonoylethanolamide.

Plasma concentration time courses of R- and S-flurbiprofen after administration of single oral doses in C57BL6/J mice (Fig 12A) revealed a percentage of R- to S-flurbiprofen inversion of approximately 23% based on AUCs from 0–8 h. Direct comparison of plasma concentrations of C57BL6/J and SJL mice (Fig 12B) with continuous oral administration in the drinking water showed equivalent bioavailability and inversion of R-flurbiprofen in these strains and equivalent circadian rhythms, which are caused by the circadian rhythms of drinking behavior. Tissue concentrations of R- and S-flurbiprofen in the periphery and brain (Fig 12C) were about 5,000 to 10,000-fold lower than plasma concentrations, which is in agreement with high plasma protein binding. Because of the inversion treatment with both R- and S-flurbiprofen reduced prostaglandins and thromboxane in the spinal cord (Fig 12D). S-flurbiprofen was somewhat more effective. Of note, only administration of S-flurbiprofen causes gastrointestinal toxicity. Interestingly, fingolimod, which was considered as a negative control in terms of prostaglandins, caused a significant increase above the levels of naïve controls.

R-flurbiprofen also modulates endocannabinoid signaling through inhibition of endocannabinoid metabolism and transport, and endocannabinoids have beneficial effects in EAE models and human MS (Loria *et al*, 2010). Therefore, endocannabinoids were analyzed in plasma, spinal cord (not shown) and somatosensory cortex (SSC). As expected from previous studies, R-flurbiprofen increased ethanolamide endocannabinoids, AEA, OEA and PEA in plasma after injection of a single dose of 10 mg/kg i.p. in C57BL6/J mice during the flare of EAE. The effect was maintained for 8 h (Fig 12E). Continuous oral R-flurbiprofen treatment in C57BL6/J mice with EAE caused an increase of all endocannabinoids in the SSC (Fig 12F). EAE per se did not alter AEA, OEA or PEA in the brain but increased 2-AG in the cortex and the spinal cord (not shown).

## Discussion

**Figure 13:**
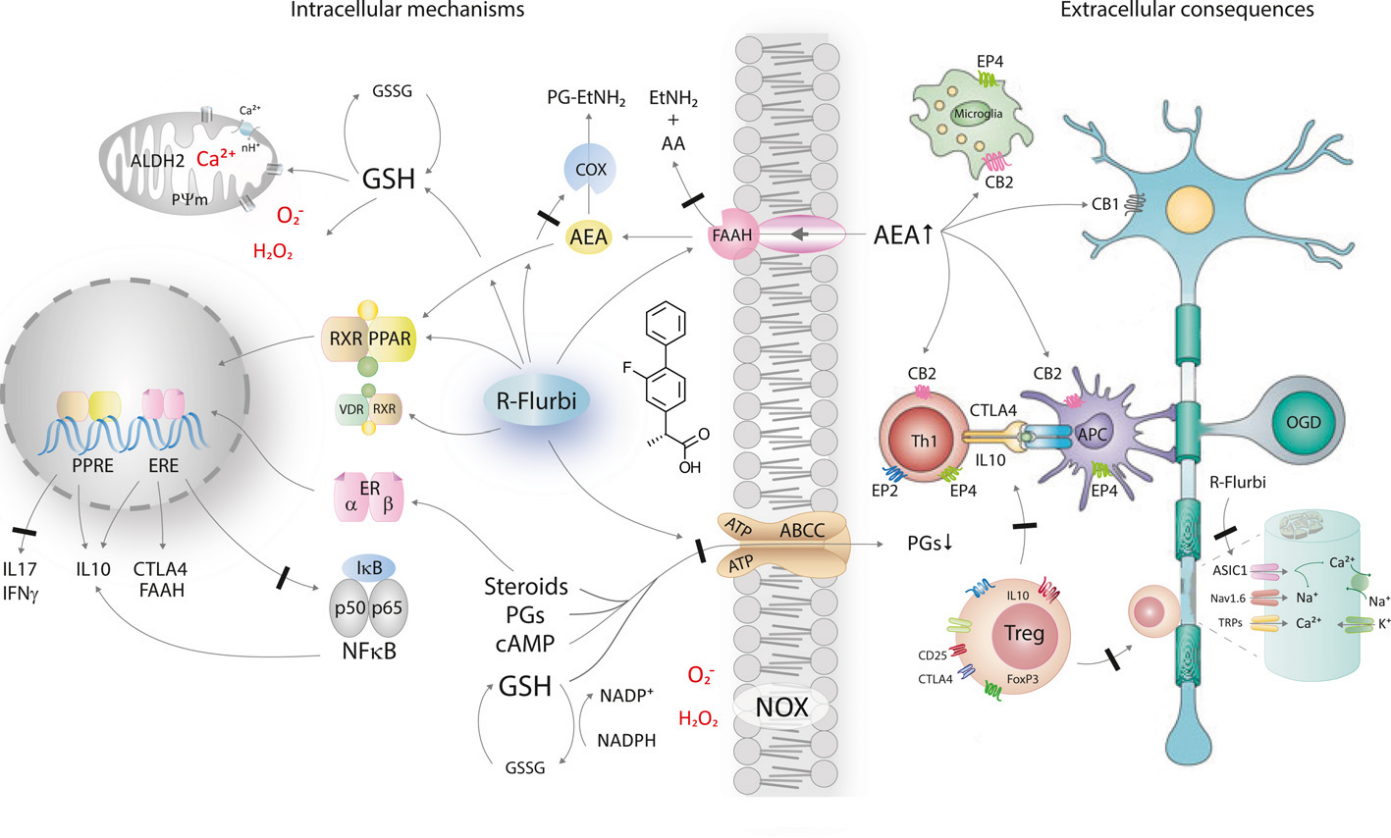
Illustration of R-flurbiprofen's mechanisms R-flurbiprofen reduces hydrolysis and cellular uptake of anandamide (AEA) by fatty acid amide hydrolase (Bishay *et al*, 2010; Holt & Fowler, 2003) and also inhibits cyclooxygenase-mediated oxidative AEA hydrolysis (Duggan *et al*, 2011) with a resulting facilitation of actions of AEA at cannabinoid CB1 and CB2 receptors and stronger intracellular AEA-mediated activation of nuclear receptors, PPARγ and PPARα (Bishay *et al*, 2010). Fortification of AEA likely further involves actions at orphan G-protein coupled receptors including GPR18 [migration of microglia (McHugh, 2012)] and GPR55 [blood-brain barrer (Waldeck-Weiermair *et al*, 2008)]. R-flurbiprofen also directly activates PPARs and RXR (Bernardo *et al*, 2005; Bishay *et al*, 2010) with a resulting increase of anti-inflammatory gene expression and repression of pro-inflammatory genes. R-flurbiprofen inhibits ATP binding cassette efflux transporters including ABCC1 and ABCC4, which transport glutathione, prostaglandins, cyclic nucleotides (cAMP and cGMP) and conjugated steroids (Bai *et al*, 2004; Brechbuhl *et al*, 2009; Keppler, 2011; Morrow *et al*, 2006; Reid *et al*, 2003; Russel *et al*, 2008). Efflux inhibition results in an attenuation of prostaglandin release (Geisslinger *et al*, 2000) but facilitation of intracellular steroid-receptor-mediated effects with an induction of CTLA4 and IL10 and repression of NF-κB and AP-1 (Tegeder *et al*, 2001), important mechanisms which contribute to the estrogen-receptor-mediated protection in EAE models (Crawford *et al*, 2010; Khalaj *et al*, 2013; Kumar *et al*, 2013; Luna *et al*, 2010; Saijo *et al*, 2011; Wu *et al*, 2013). Blocking the efflux of glutathione will increase the intracellular glutathione pool and increase the antioxidative capacity, which may explain previously observed reductions of lipid peroxidation (Lopez-Villodres *et al*, 2011), and prevention of mitochondrial calcium overload in stressed cells (Sanz-Blasco *et al*, 2008) likely contributed by increasing chaperone activity and reduction of ER stress (Hosoi *et al*, 2014). Collectively, these effects favor the differentiation of regulatory and inflammation-resolving T cells. R-flurbiprofen also inhibits hydrogen sensitive ASIC1 channels (Mishra *et al*, 2010; Voilley *et al*, 2001), which contribute to pathological firing of demyelinated axons, and ASIC1 inhibition is neuroprotective in EAE models (Arun *et al*, 2013; Friese *et al*, 2007).

R-flurbiprofen prevented EAE when administered early after immunization and reduced RR-EAE if treatment was initiated late, after completion of the first flare. Efficacy was shown for clinical scores, microglial activation, macrophage and T-cell invasion and gene regulation by *in vivo* imaging, histology, quantitative FACS analyses and microarray gene expression analysis. The mechanisms likely involve a combination of the previously described molecular effects of R-flurbiprofen (illustrated in Fig 13), including modulation of endocannabinoids (Bishay *et al*, 2010; Duggan *et al*, 2011; Holt & Fowler, 2003), activation of PPARs (Bernardo *et al*, 2005; Bishay *et al*, 2010) and retinoid X receptor (RXR) (You *et al*, 2009), inhibition of NF-κB and AP1 (Tegeder *et al*, 2001) and of acid sensitive ion channels (ASIC1) (Mishra *et al*, 2010; Voilley *et al*, 2001) and propagation of antioxidative capacity (Lopez-Villodres *et al*, 2011; Sanz-Blasco *et al*, 2008). PPAR activators and inhibitors of NF-κB are currently being evaluated as potential therapeutics for MS (Dasgupta *et al*, 2007; Dunn *et al*, 2010; Klotz *et al*, 2005) mainly because they reduce the inflammatory component of the disease. R-flurbiprofen's effects are weaker than those of specific drugs, but R-flurbiprofen likely provides additional protection of neurons and oligodendrocytes through its antioxidative effects and inhibition of ASIC1, which has been identified as another promising therapeutic target in EAE models (Arun *et al*, 2013). The combination of the favorable molecular mechanisms (Fig 13) likely drives the immune system toward immune tolerance and resolution of inflammations as reflected by the observed favoring of Tregs and CD152/CTLA4 positive T cells, which down-regulate an activated immune system (Venken *et al*, 2010; Walker, 2013) and hence stop autoaggressive T cells from destroying oligodendrocytes and myelin (Racke *et al*, 2000; Venken *et al*, 2010). Tregs are crucial for the maintenance of immune tolerance, and disturbances of Tregs in multiple sclerosis contribute to disease progression and relapse (Racke *et al*, 2000; Venken *et al*, 2010). The observed favoring of these cell subpopulations by R-flurbiprofen therefore likely helps to attenuate the pathologically activated immune system. In addition, R-flurbiprofen favors microglia with a resting phenotype, which likely provide an environment favoring remyelination (Voss *et al*, 2012).

During the onset of EAE, the effects may also involve inhibition of prostaglandin synthesis, which in mice is a consequence of R- to S-flurbiprofen inversion and thus S-enantiomer-mediated cyclooxygenase inhibition. The effect may vary depending on the receptors. For example, PGE_2_ promotes immunization through EP2 and EP4 receptors on Th1 and Th17 cells, but PGE_2_ also blocks T-cell infiltration of the CNS by acting on EP4 receptors of endothelial cells at the blood-brain barrer (Esaki *et al*, 2010). Prostaglandins are unlikely to be major players in the pathogenesis of human multiple sclerosis, as suggested by the ineffectiveness of non-steroidal anti-inflammatory drugs in terms of relapse frequency or progression, but particularly COX-1, which is mainly expressed in microglia (Maihofner *et al*, 2000), might be a driver of relapse in mice as suggested by its upregulation during relapses (Ayoub *et al*, 2011).

The inversion of R- to S-flurbiprofen in mice amounted to approximately 25%, but oral treatment with S-flurbiprofen was not 3-4-fold more effective in terms of PG inhibition, suggesting that R-flurbiprofen additionally reduces prostaglandins independently of cyclooxygenases, likely by inhibiting their cellular efflux by the ATP binding cassette transporter, MRP4/ABCC4 (Reid *et al*, 2003). Various non-steroidal anti-inflammatory drugs, including racemic flurbiprofen inhibit this transporter (Reid *et al*, 2003), and also block effluxes mediated by ABCC1 and ABCC2 (El-Sheikh *et al*, 2007; Keppler, 2011) and organic anion transporters (Honjo *et al*, 2011; Mulato *et al*, 2000). Besides prostaglandins, ABCC transporters accept cyclic nucleotides (Keppler, 2011; Russel *et al*, 2008), steroids (Chu *et al*, 2004) and glutathione (Rebbeor *et al*, 2000; Salerno *et al*, 2004) as cargo and inhibition of the efflux raises intracellular levels of these molecules. The observed gene regulations with the strong increase of CTLA4 and IL-10 under R-flurbiprofen treatment suggest a facilitation of steroid, possibly estrogen receptor signaling conferring protection in EAE through induction of anti-inflammatory genes and transrepression of pro-inflammatory genes (Kumar *et al*, 2013; Morales *et al*, 2006; Offner, 2004; Saijo *et al*, 2011).

The previously observed PPAR activating and NF-κB inhibiting functions of R-flurbiprofen also suggested that its therapeutic effects in EAE may be brought about by differential gene regulation. Indeed, microarray analysis identified several genes, which were up or down-regulated exclusively in vehicle EAE, or were more strongly regulated in the vehicle group. The differences partly reflect the differences in the severity of the disease but the tissue was taken from score-matched pairs as far as possible so that the differences also point to specific effects of R-flurbiprofen. The ontology annotations of the genes, which differed most strongly between R-flurbiprofen and vehicle, pointed toward ‘cell adhesion’ and ‘receptor binding’ as potential processes. Some of the differentially regulated genes, such as the IL-7 receptor (Ilr7) (Gregory *et al*, 2007), tumor necrosis factor receptor superfamily 12, TNFRSF12/DR3 (Pappu *et al*, 2008) and TNF alpha induced protein 3 (TNFAIP3) (Prahalad *et al*, 2009) are known to confer susceptibility to multiple sclerosis. In addition, other candidates, such as chitinase-3-like (Chi3 l) (Canto *et al*, 2012; Comabella *et al*, 2010) and soluble TLR-4 co-receptor, sCD14 (Brettschneider *et al*, 2002) have been evaluated as biomarkers for MS, suggesting that R-flurbiprofen regulated several genes which are important in the human disease. Importantly, R-flurbiprofen *per se* did not affect gene expression in CFA control mice, which would be indicative of immunosuppressive effects but apparently, it blocks the activation process both of microglia/macrophages and of T cells and may help to maintain immune tolerance.

This silencing of the immune system may be contributed by an increase of endogenous cannabinoids and consequently facilitation of CB2 signaling. R-flurbiprofen inhibits both FAAH- and cyclooxygenase-mediated metabolism of endocannabinoids and the effect is independent of R to S inversion and COX-mediated prostaglandin synthesis (Duggan *et al*, 2011). COX-1 is highly expressed in the somatosensory cortex, that is, at the site where R-flurbiprofen caused the strongest increase of endocannabinoids. A facilitation of CB1 signaling at this site may well explain the antinociceptive effects and may be contributed by peripheral effects on CB1 receptors of nociceptors (Agarwal *et al*, 2007). A single i.p. injection of R-flurbiprofen during the flare of EAE caused a strong rise of ethanolamide endocannabinoids in plasma, which does not occur in mice without EAE (Bishay *et al*, 2013) suggesting that EAE increases the endocannabinoid-rising capacity of R-flurbiprofen.

In conclusion, R-flurbiprofen probably does not have one very specific, clearly defined target and effects of R-flurbiprofen in mice may be contributed by the S-enantiomer-mediated inhibition of prostaglandin synthesis. Nevertheless, R-flurbiprofen likely combines multiple effects at molecular targets independently of prostaglandins, which are currently being evaluated as therapeutic targets in MS, such as the PPARs (Dunn *et al*, 2010; Lovett-Racke *et al*, 2004; Xu & Drew, 2007), NF-κB (Mc Guire *et al*, 2013; van Loo *et al*, 2006) and p38 MAPK (Kleinewietfeld *et al*, 2013). The drug may therefore combine mild anti-inflammatory, immune modulatory and anti-oxidative properties with low toxicity. Since the inversion from R to S is lower in humans than in mice, it remains to be answered to what extent R-flurbiprofen may exert efficacy in multiple sclerosis. Its low toxicity may justify a clinical evaluation.

## Materials and Methods

### Animals and drug treatments

Female C57BL6/J and female SJL mice (Charles River and Harlan Winkelmann, Germany), aged 10–12 weeks at immunization, were used for study of primary progressive EAE and relapsing-remitting EAE, respectively. Mice were housed at 3-5 mice per cage at constant room temperature (21 ± 1°C) under a regular light/dark schedule with light from 7:00 a.m. to 7:00 p.m. Food and water were available *ad libitum*. Animals were treated orally with R-flurbiprofen (Aesica, UK), S-flurbiprofen (Cayman Chemical) or vehicle or FTY720 (Cayman Chemical) via the drinking water. FTY720 (fingolimod) was used as the positive control at 0.5 mg/kg/day. The therapy was continuous and started on day 3 after immunization for preventive treatment, on day 7–8 to allow for some immune activation for analysis, 4 days before onset of clinical symptoms for semi-therapeutic treatment (C57BL6/J), on day 13 after full development of EAE for late-therapeutic treatment of C57BL6/J mice or after the first peak of the disease 19 days after immunization for late-therapeutic treatment of SJL mice. For late-therapeutic treatment of C57BL6/J mice that have a primary progressive course of the disease and do not recover, R-flurbiprofen or vehicle were administered via drug or vehicle soaked sweet cornflakes to ensure drug, fluid and calories intake during the disease. The animals were accustomed to the cornflakes before the start of the therapy. The evaluation of these different therapeutic paradigms increases the predictability of a potential clinical usefulness of R-flurbiprofen in human MS. For the ‘late treatment’, mice were allocated pairwise to vehicle and R-flurbiprofen groups according to their clinical scores during the first peak so that the scores were identical in both groups at the onset of treatment. The doses of R-flurbiprofen were 2.5, 5 and 10 mg/kg in C57BL6/J mice and 5 mg/kg/day for SJL mice. S-flurbiprofen was used at 10 mg/kg/day. The purity of R- and S-flurbiprofen was > 99.9%, and the stability in drinking water and food was confirmed by LC-MS/MS analyses for up to 7 days at room temperature. After this time, recovery of R-flurbiprofen was 95.7% and of S-flurbiprofen 91.5%. The experiments adhered to the guidelines of the Committee for Research and Ethical Issues of the International Association for the Study of Pain (IASP) and to those of GV-SOLAS for animal welfare in science. They were approved by the Local Ethics Committee for Animal Research (Darmstadt, Germany).

### EAE model

C57BL6/J mice were immunized according to a standard protocol using the Hooke Kit™ MOG35-55/CFA emulsion PTX (EK-2110, Hooke Labs, St Lawrence, MA), which contains 200 μg myelin oligodendrocyte glycoprotein (MOG) 35–55 emulsified in 200 μl Complete Freund's Adjuvant (CFA). The emulsion was injected subcutaneously at two sites followed by two intraperitoneal (i.p.) injections of 200 ng pertussis toxin (PTX) in phosphate buffered saline (PBS), the first 1–2 h after MOG35-55, and the second 24 h after MOG35-55. Control mice received CFA without MOG35-55. SJL mice were immunized according to a standard protocol using Hooke Kit™ PLP139-151/CFA emulsion PTX (EK-0123), which contains 200 μg myelin proteolipid protein (PLP) 139–151 in 200 μl CFA (Hooke Labs, US). The emulsion was injected subcutaneously at two sites followed by two i.p. injections of 200 ng PTX in PBS, the first 1–2 h after PLP135-151, and the second 24 h after PLP135-151. Nociception was analyzed before onset and during the course of the disease in remission periods. EAE scores were daily assessed to evaluate the severity and extent of motor function deficits: score 0, no obvious changes in motor functions; score 0.5, distal paralysis of the tail; score 1, complete tail paralysis; score 1.5, mild paresis of one or both hind legs; score 2, severe paresis of hind legs; score 2.5, complete paralysis of one hind leg; score 3, complete paralysis of both hind legs; score 3.5, complete paralysis of hind legs and paresis of one front leg. Mice reaching a score of 3.5 were killed. Locomotion and coordination was further analyzed with the Rota Rod test (Ugo Basile) in the score-free first remission in SJL mice, 20 days after immunization. Two hours before the tests, mice were trained for 2 min. The rod was rotating at a constant speed of 16 rpm. The fall-off latency was averaged from three tests, and the cutoff time was 120 s.

For bone marrow transplantation (BMX), recipient C57BL6/J mice received a 9.5 Gy cobalt 60 gamma irradiation and subsequently an intravenous injection through the tail vein of 6 × 10^6^ bone marrow cells, which were harvested from tibia and femur of β-actin-EGFP donor mice. Immunization was done with MOG35-55/CFA emulsion plus PTX (protocol C57BL6/J) 3 weeks after BMX and treatment with R-flurbiprofen or vehicle started 3 days after immunization.

### Behavioral analysis of nociception

Behavioral tests were performed without knowledge of the genotype. After habituation to the testing cages, mice were tested for their reaction latencies to mechanical, cold and heat stimulation. A Dynamic Plantar Aesthesiometer (Ugo Basile, Italy) was used to assess mechanical nociception, whereby a von Frey-like filament is pushed against the plantar hind paw with linear ascending force (0–5 g at 0.5 g/s) and then maintained at 5 g until a strong and immediate withdrawal occurs. The paw withdrawal latency was the mean of three consecutive trials with intervals of at least 30 s. The acetone test was used to measure cold allodynia. After application of a drop of acetone to the plantar hind paw, mice lick, lift and shake the nerve injured paw. These reactions were monitored with a stop watch for 90 s, starting immediately after application of acetone. Heat hyperalgesia was assessed by recording the paw withdrawal latency on a hot plate at 52°C or in the Hargreaves test, in which a radiant heat source is placed underneath one hind paw or underneath defined points of the tail and heat application is started with a button. The heating is automatically stopped upon paw or tail withdrawal, and the latency is monitored. Three tests at intervals of at least 5 min were performed and the results averaged. Nociceptive behavior was assessed before immunization, so that each animal had its own baseline value, before onset of clinical EAE scores, in the first and second remission.

### Quantitative RT–PCR and microarray analysis

Total RNA was extracted from homogenized tissue according to the protocol provided in the RNeasy tissue Mini Kit (Qiagen, Hilden, Germany), reverse transcribed using 500 ng RNA as template and oligo-dT and Random Hexamers (Ration 1:2) as primers to obtain cDNA fragments. Quantitative real-time PCR was performed using the SYBRgreen detection system with primer sets designed using Primer Express. Transcript regulation was determined using the relative standard curve method according to the manufacturer's instructions (Applied Biosystems).

For microarray analysis, total RNA was checked for quality (Nanodrop ND-1000, Agilent 2100 Bioanalyzer), subsequently biotinylated and hybridized to Mouse Sentrix-6 V2 Expression BeadChips (Illumina), which targets approximately 45,000 mouse transcripts and variants and provides comprehensive coverage of the transcribed mouse genome on a single array. It enables the interrogation of six samples in parallel. Each sample consisted of pooled lumbar spinal cord tissue from 3 animals. Three samples were analyzed per group. Groups were CFA control with vehicle treatment, CFA control with R-flurbiprofen, EAE-vehicle and EAE-R-flurbiprofen treatment. Treatment was started 5 days after immunization. For dissection, pairs were matched according to the clinical scores. QC, labeling, hybridization and raw data evaluation and normalization were done according to standard protocols at the core facilities of the Deutsche Krebsforschungszentrum (DKFZ), Heidelberg, Germany. The microarray data from this publication have been submitted to the GEO database (http://www.ncbi.nlm.nih.gov/geo/) and assigned the identifier accession number GSE60847.

### Enzyme immuno assay for myelin basic protein (MBP)

Brain and spinal cord tissue were homogenized in PhosphoSafe extraction buffer (Sigma) containing a protease inhibitor cocktail (Roche) and PMSF 10 μg/ml followed by centrifugation. Total extracted proteins were quantified with the Bradford method. The MBP ELISA (Elabscience) was done according to the instructions of the manufacturer, and results were normalized per mg of total protein.

### FACS analysis of surface marker proteins

Single cell suspensions were prepared from the spleen and the lumbar spinal cord segment. The L4-L6 spinal cords were rapidly dissected, treated with lysis buffer (DMEM/Accutase (PAA) 1:1, collagenase (3 mg/ml, Sigma), DNAse I (1 U/ml, Promega)) for 45 min at 37°C and subsequent mechanical disruption, which was done by forcing the tissue through a nylon mesh with 70 μm pore size (Cell Strainer, BD). Cells were then treated with erythrocyte lysis buffer for 10 min at room temperature and CD16/32 blocking antibody (Fcγ RII/III receptor blocker, BD) for 15 min on ice. For staining of cell surface antigens, cells were incubated for 20 min at room temperature in staining buffer with the respective fluorochrome labeled antibodies, as listed in Supplementary Table S1a, and were then counted with a flow cytometer (BD FACS Canto II). FACS scans of score-matched mice were analyzed with FlowJo 10.06. The antibody concentrations were as recommended by the manufacturers, and the controls were FITC, PE, or APC-conjugated rat IgG.

### FACS analysis of intracellular cytokines

Protein expression of intracellular cytokines was assessed by FACS analysis. Singe cell suspensions were prepared from the spleen by mechanical disruption by forcing the tissue through a nylon mesh with 70 μm pore size (Cell Strainer, BD), and the pellets were resuspended in PBS with 10% fetal calf serum (FCS). Cells were then stimulated with 50 ng/ml phorbol 12-myristate 13-acetate (PMA) and 500 ng/ml ionomycin (Sigma) for 8 h at 37°C. After 2 h, 10 μg/ml brefeldin A (Sigma) was added to disrupt the structure and function of the Golgi apparatus. The stimulated cells were then treated with erythrocyte lysis buffer for 10 min at room temperature and incubated overnight in fixation/permeabilization buffer (Becton Dickinson) at 18°C. For staining of cell surface antigens and intracellular cytokines, cells were incubated for 20 min at room temperature in permeabilization buffer with the respective fluorochrome labeled antibodies as listed in Supplementary Table S1a and were then counted with a flow cytometer (BD FACS Canto II) and analyzed with FlowJo 10.06.

### Immunofluorescence

Terminally anesthetized mice were transcardially perfused with ice cold 0.9% saline followed by 4% paraformaldehyde (PFA) in 0.1 M phosphate buffered saline (PBS, pH 7.4). The L4 and L5 spinal cord segments and the optic nerve were dissected, post-fixed for 2 h in PFA and transferred into sucrose (20% in PBS) for overnight cryoprotection at 4°C. The tissue samples were embedded in Tissue-Tek® O.C.T. Compound (Science Services, Munich, Germany), cut into 14 μm transverse sections on a cryotome and mounted on glass slides. Sections were permeabilized for 20 min in PBST (0.1% Triton X-100 in PBS), blocked for 30 min with 3% bovine serum albumin (BSA) in PBST and incubated overnight at 4°C with primary antibodies (Supplementary Table S1b) dissolved in 1% BSA in PBST. After washing in PBST, sections were incubated for 2 h at room temperature with species-specific secondary antibodies conjugated with Alexa Fluor 488 or 549 (Invitrogen, Karlsruhe, Germany) or Cy3 (Sigma-Aldrich, Munich, Germany). After immunostaining, slides were rinsed in PBS and cover-slipped in Fluoromount G (Southern Biotech, Birmingham, AL). Images were obtained using a Zeiss Axiovert fluorescent microscope and analyzed with AxioVision 4.7 software (Zeiss).

### *In vivo* imaging of brain inflammation and optic neuritis

*In vivo* imaging was done with an IVIS Lumina Spectrum, which allows for analysis of bioluminescence and near-infrared signals, which were analyzed with LivingImage software (Perkin Elmer). Brain inflammation and optic neuritis and leakage of the blood-brain barrer were assessed at the first peak of the disease or in the score-free second remission in SJL mice. R-flurbiprofen treatment started 3 or 5 days after immunization or during the first remission 19 days after immunization. Images of 5–10 mice were captured per group and analyzed. During all imaging procedures, mice were kept under 1–1.5% isoflurane anesthesia.

Encephalitis and optic neuritis were assessed with the bioluminescent XenoLight RediJect Inflammation Probe (Perkin Elmer), which is a chemiluminescent reagent in a ready-to-use format (40 mg/ml) that allows for *in vivo* assessment of MPO levels, and with near-infrared MMPsense-680 (Perkin Elmer), which is bio-activated by metalloproteinases at sites of inflammation. XenoLight RediJect Inflammation Probe (100 μl) was injected intraperitoneally, and bioluminescence was captured 5, 10 and 15 min after injection. The IVIS settings were Epi-BLI, Em filter open, Ex filter block, fstop 1, binning 8, focus B 6.5 cm, exposure 120 s. For each mouse, the two maximum time points for total counts of bioluminescence signals were used for statistical analysis. Non-responder mice without symptoms of EAE were used as imaging controls and gave no signal. MMPsense-680 (2 nmol/150 μl in 0.1 M PBS) was injected intravenously 24 h before imaging. The IVIS settings were Epi-FL, Ex640/Em700, Ex680/Em720, exposure 1 s, focus B 6.5 cm, binning 8, fstop 2. Spectral unmixing was performed with autofluorescence images, and the unmixed images were used for quantitative analysis of the total radiant efficiency, which is implemented in LivingImage.

Leakage of the blood-brain barrer was assessed with bovine serum albumin coupled with Cy5.5 (BSA-Cy5.5.), which distributes very slowly from the blood into the interstitial space except for sites of inflammation, where the dye accumulates. BSA-Cy5.5 (50 mg/kg) was injected i.v., and imaging was done 2–3 h after injection. The settings were transillumination-FL, 2 sites, Ex640/Em700, Ex680/Em720, exposure 1 min, binning 8, fstop 2, focus B 6.5 cm.

Myelin imaging was performed with 3,3-diethylthiatricarbocyanine iodide (DBT), a near-infrared dye that binds to myelin and can be used to visualize and quantify cuprizone-induced demyelination (Wang *et al*, 2011) or EAE-evoked myelin inflammation. After taking a baseline image, DBT 0.3 mg/kg in 100 μl 0.1 M PBS with 5% DMSO was injected intravenously through the tail vein and images were captured 2 and 4 min after injection. The settings were Epi-Fl, Ex745/Em800, exposure 2 s, focus B 6.5 cm, binning 8, fstop2. Total radiant efficiency of automatically detected ROIs was used for quantification.

### Analysis of endocannabinoids and prostaglandins

Lipid analysis was performed using liquid chromatography-electrospray ionization-tandem mass spectrometry (LC-ESI-MS/MS). The LC-MS/MS system consisted of a hybrid triple quadrupole-ion trap QTrap 5500 mass spectrometer (AB Sciex, Darmstadt, Germany) equipped with a Turbo-V-source operating in negative ESI mode, an Agilent 1200 binary HPLC pump, column oven (40°C) and degasser (Agilent, Waldbron, Germany) and an HTC Pal autosampler (Chromtech, Idstein, Germany) with a cooling stack which kept the samples at 4°C. High purity nitrogen for the mass spectrometer was produced by a NGM 22-LC/MS nitrogen generator (cmc Instruments, Eschborn, Germany).

### Analysis of endocannabinoids

Analysis of anandamide (AEA), palmitoylethanolamide (PEA), 1 and 2-arachidonoylglycerol (1+2-AG) and oleoylethanolamide (OEA) was done as described (Bishay *et al*, 2013). Briefly, tissue pieces of approximately 2 mg were weighed, homogenized and extracted by liquid-liquid extraction, and the reconstituted samples were analyzed for endocannabinoids. The respective deuterated substances AEA-d8, PEA-d4, 2-AG-d5, 1-AG-d5 and OEA-d2 were used as internal standards.

Two cycles of liquid-liquid extraction were performed. Ethylacetate: n-hexane (50 ml 9:1, v/v) was added to the tissue homogenate or to a 50 μl plasma sample, spiked with the corresponding internal standard, vortexed and centrifuged for 3 min at 17,000 × *g*. The organic phase was removed, and the extraction repeated with 150 ml extraction solvent. The organic fractions were combined and evaporated at a temperature of 45°C under a gentle stream of nitrogen. The residues were reconstituted with 50 μl of acetonitrile in glass vials, and 10 μl was injected into the LC-MS/MS system.

HPLC analysis was done under gradient conditions using a Luna C18 column (150 mm L × 2 mm ID, 5 μm particle size, Phenomenex, Aschaffenburg, Germany). Precursor-to-product ion transitions of *m/z* 346→259 for AEA, *m/z* 354→267 for AEA-d8, *m/z* 298→268 for PEA, *m/z* 302→272 for PEA-d4, *m/z* 377→303 for 2-AG and 1-AG, *m/z* 382→303 for 2-AG-d5 and 1-AG-d5, *m/z* 324→86 for OEA and *m/z* 326→86 for OEA-d2 were used for the multiple reaction monitoring (MRM) with a dwell time of 50 ms.

### Analysis of prostaglandins

The homogenized tissue pieces were extracted by two rounds of liquid-liquid extraction with ethylacetate (600 μl). The samples were spiked before extraction with the internal standard mixture, 100 μl 0.15 M EDTA and 10 μl BHT (2 mg/ml in methanol) to prevent oxidation of analytes. After vortexing and centrifugation for 3 min at 17,000 × *g*, the organic fractions were combined and evaporated at a temperature of 45°C under a gentle stream of nitrogen.

LC-MS/MS conditions: For the chromatographic separation, a Synergi Hydro-RP column and pre-column were used (150 × 2 mm I.D., 4-μm particle size and 80 Å pore size from Phenomenex, Aschaffenburg, Germany). A linear gradient was employed at a flow rate of 300 μl/min. The mobile phase A was water/formic acid (100:0.0025, v/v, pH 4.0) and mobile phase B acetonitrile/formic acid (100:0.0025, v/v). The sample solvent was acetonitrile/water/formic acid (20:80:0.0025, v/v, pH 4.0). The total run time was 16 min and injection volume 45 μl. Retention times of 6-keto-PGF_1α_, TXB_2_, PGF_2α_, PGE_2_ and PGD_2_ were 7.4 min, 8.0 min, 8.2 min, 8.7 min and 9.2 min, respectively.

The mass spectrometer was operated in the negative ion mode with an electrospray voltage of -4500 V at 450°C. Multiple reaction monitoring (MRM) was used for quantification. The mass transitions used are *m/z* 351.1 →*m/z* 315.0 for PGE_2_ and PGD_2_, *m/z* 353.1 →*m/z* 309.2 for PGF_2α_, *m/z* 369.1 →*m/z* 163.0 for 6-keto-PGF_1_ , *m/z* 369.1 →*m/z* 169.1 for TXB_2_, *m/z* 355.1 →*m/z* 275.1 for [^2^H_4_]-PGE_2_ and [^2^H_4_]-PGD_2_, *m/z* 357.1 →*m/z* 313.2 for [^2^H_4_]-PGF_2_ , *m/z* 373.2 →*m/z* 167.1 for [^2^H_4_]-6-keto-PGF_1α_ and *m/z* 373.1 →*m/z* 173.1 for [^2^H_4_]-TXB_2_ all with a dwell time of 50 ms. All quadrupoles were working at unit resolution.

### Quantification

Concentrations of the calibration standards, quality controls and unknowns were evaluated by Analyst software (version 1.5; B Sciex, Darmstadt, Germany). Ratios of peak areas of the lipid and the respective internal standard (*y*-axis) were plotted against concentration (*x*-axis), and calibration curves for each lipid were calculated by least square regression analysis with 1/concentration^2^ weighting. Variations in accuracy and intra-day and inter-day precision (*n* = 6 for each concentration, respectively) were < 15% over the respective ranges of calibration.

### Analysis of R- and S-flurbiprofen

Fifty microliter plasma or homogenized tissue was spiked with 50 μl acetonitrile, 20 μl of the internal standard solution (250 ng/ml R/S-flurbiprofen-d5 in methanol) and 300 μl acetonitrile, in order to precipitate the proteins. After vortexing and centrifugation, 150 μl of the supernatant was transferred to a glass vial and 20 μl of this solution was injected into the LC-MS-MS system, which consisted of a high performance liquid chromatography system (Agilent, Waldbronn, Germany) coupled to a tandem mass spectrometer. HPLC analysis was done under gradient conditions using a Lux Cellulose-3 column (150 × 2 mm, 3 μm) (Phenomenex, Aschaffenburg, Germany) and water and acetonitrile containing 0.0025% formic acid. MS/MS analyses were performed on an API 5000, a triple quadrupole mass spectrometer with a turbo ion spray source operated in the negative ion mode. Precursor-to-product ion transitions of *m/z* 243.0→199.0 for R- and S-flurbiprofen and *m/z* 248.1→204.0 for R- and S-flurbiprofen-d5 (internal standard) were used for multiple reaction monitoring (MRM). Quantification was done with Analyst software 1.5 (AB Sciex, Darmstadt, Germany). Linearity of the calibration curve was proven from 0.01 to 30 μg/ml. The coefficient of correlation for all measured sequences was at least 0.99. The intra-day and inter-day variability was < 15%.

### Statistics

Statistical analysis was done with SPSS (version 21 for Windows, IBM SPSS) and Graphpad Prism 6 (Statcon), and results are presented as mean ± SEM (behavior, EAE scores) or mean ± SD (imaging, biochemical and FACS data). Time courses of EAE scores and nociceptive behavior were submitted to analysis of variance for repeated measures (rm-ANOVA) with ‘time’ as within and ‘treatment’ as between subject factors. Additionally, areas under the time versus score or withdrawal curves (AUCs) were calculated according to the linear trapezoidal rule and submitted to univariate analysis of variance. Subsequently, differences between treatment groups of individual time points were assessed by two-sided unpaired *t*-tests. Non-time course behavioral data and imaging results were analyzed by two-sided unpaired Student's *t*-tests or with univariate analysis of variance (ANOVA) in case of three groups including naïve, vehicle and R-flurbiprofen. ANOVA was followed by *post hoc* analyses using a correction of alpha according to Bonferroni or Dunnett. FACS data were analyzed by two-way ANOVA using ‘population’ as within and ‘treatment’ as between subject factors, followed by *post hoc* analyses. Similarly, lipid signaling molecules were analyzed using ‘lipid’ as within and ‘treatment’ as between subject factor. Linear regression analyses were used to assess differences in gene expression in microarray experiments and to calculate the percentage of R- to S-flurbiprofen inversion in experiments using continuous oral treatment. For plasma concentration versus time courses, AUCs were used to roughly estimate the percentage of inversion. The α level was set at 0.05 for all statistical comparisons. The number of animals used in the experiments is specified in the respective figure legends or Supplementary Tables.

## For more information:

Multiple Sclerosis Societies:

http://www.dmsg.de/

http://www.mssociety.org.uk/

http://www.nationalmssociety.org/

http://www.mymsaa.org/

http://www.mssociety.ca/

General information

http://en.wikipedia.org/wiki/Multiple_sclerosis

http://www.msfocus.org/
