# Supplementary material for: R-flurbiprofen attenuates experimental autoimmune encephalomyelitis in mice
Source: EMBO Mol Med. 2014 Sep 30;6(11):1398–422. doi: 10.15252/emmm.201404168 (PMC4237468; doi:10.15252/emmm.201404168)
Supplement: Supplementary file 1 [file emmm0006-1398-sd1.pdf]

Manuscript EMM-2014-04168

R-flurbiprofen attenuates experimental autoimmune encephalomyelitis in mice

Katja Katja Schmitz, Natasja deBruin, Philipp Bishay, Julia Männich, Annett Häussler, Christine Altmann, Nerea Ferreirós, Jörn Lötsch, Alfred Ultsch, Michael J. Parnham, Gerd Geisslinger, Irmgard Tegeder

Corresponding author: Irmgard Tegeder, Goethe University Frankfurt

Review timeline:

Submission date:	14 April 2014
Editorial Decision:	12 May 2014
Revision received:	04 August 2014
Editorial Decision:	21 August 2014
Revision received:	28 August 2014
Accepted:	29 August 2014

Transaction Report:

(Note: With the exception of the correction of typographical or spelling errors that could be a source of ambiguity, letters and reports are not edited. The original formatting of letters and referee reports may not be reflected in this compilation.)

Editor: Céline Carret

1st Editorial Decision 12 May 2014

Thank you for the submission of your manuscript to EMBO Molecular Medicine. We have now heard back from the two referees whom we asked to evaluate your manuscript. Although the referees find the study to be of potential interest, they also raise a number of concerns, and make suggestions to render the study more attractive and compelling to our readership.

As you will see from the below reports, while referee 1 is rather positive about the study, referee 2 is much more critical and makes several important points that you should take into account in your revised manuscript as we believe it would greatly improve the current study. I would like to stress that with limited mechanism, a strong medical impact would be required for the manuscript to be accepted. As such, I would like to strongly encourage you to follow referee 2's recommendations.

Should you address these comments satisfactorily, we would welcome the submission of a revised version for further consideration and depending on the nature of the revisions, this may be sent back to the referees for another round of review.

Please note that it is EMBO Molecular Medicine policy to allow a single round of revision in order to avoid the delayed publication of research findings. Consequently, acceptance or rejection of the manuscript will depend on the completeness of your responses included in the next version of the

manuscript.

EMBO Molecular Medicine has a "scooping protection" policy, whereby similar findings that are published by others during review or revision are not a criterion for rejection. Should you decide to submit a revised version, I do ask that you get in touch after three months if you have not completed it, to update us on the status.

Please also contact us as soon as possible if similar work is published elsewhere. If other work is published we may not be able to extend the revision period beyond three months.

I look forward to seeing a revised form of your manuscript as soon as possible.

***** Reviewer's comments *****

Referee #1 (Comments on Novelty/Model System):

Data are of a high quality and the study may have important medical impact. There are no major issues to address.

Referee #1 (Remarks):

This is an excellent manuscript in which the effects of R-flurbiprofen as a potential therapeutic agent for multiple sclerosis are explored in EAE models in mouse. Multiple models and clinically relevant time points for the effectiveness of R-fluriprofen are measured, and numerous readouts at the behavioral level (nociceptive testing), cellular level (immune cell infiltration, microglial activation etc), and molecular level (microarray analysis) are used to monitor disease progression and potential mechanisms. The authors demonstrate the R-flurbiprofen has strong effects and have gone to some effort to understand the mechanisms of its actions. While they have not been able to discover a single mechanism, they have explored multiple options, and in light of the literature conclude that a combination of mechanisms is probably responsible. In summary, this appears to be an important study and as pointed out by the authors, their data strongly supports further clinical investigations of R-flurbiprofen in multiple sclerosis.

Minor points:
Please explain why the hot plate test and Dynamic Plantar Aesthesiometer tests were used in C57BL6 mice, and Hargreaves test and tail-flick were used in SJL mice.
Use "resting" rather than "peaceful" when describing microglial phenotypes.

Referee #2 (Comments on Novelty/Model System):

Technologies and model system applied are adequate. Novelty is high (to my knowledge this is the first time that R-flubibrofen has been found to be of any therapeutic usefulness rather than being viewed as the inactive isomer of s-flurbiprofen). However, medical impact is medium at the best because compound is very effective in the RR form of MS and much less in the progressive form, which is, indeed, the area with major medical need (the RR form of MS has already some effective medications.

Referee #2 (Remarks):

This is a potentially interesting study but provides only description of effects without a real interpretation of the findings. Based on data presented, R-flurbiprofen effect in MS is the

consequence of its simultaneous action on several targets, none of which is really decisive. Only the sum of each single contribution gives the final effect.
To become really attractive to the reader, paper needs to be improved. Some comments/suggestions could be the following:
ïIn the current format findings are entirely linked to the peculiar pharmacological profile of R-flubiprofen. In my view, authors should try to identify if a main molecular target is primarily engaged and drives the efficacy in MS more than the other targets. Then the focus of research (and discussion) should be on this mechanism.
ïData on R-Flubiprofen in EAA induced demyelination should be provided. A simple measure of MBP could be sufficient. This will strength the concept that myelin sheath destruction is reduced/halted by R-flubiprofen treatment
ïNot clear the extent at which R-flubiprofen multi-targets are shared by the S-form. Could be helpful to make comparative studies since they might unmask MS related specific targets.
ïNot clear the contribution of the micro array study. As expected several genes were found up-regulated, others were found down-regulated and the expression of others did not change. Authors should better discuss the impact of these findings (favorite option) or , if not impact, withdraw them.
ïAuthors found that R-flubiprofen can prevent/reduce EAE development in C57 mice (model of progressive MS). Wondering if drug can also reverse established symptoms in diseased animals (curative protocol). This would provide much greater medical impact.

1st Revision - authors' response 04 August 2014

Thank you for evaluating our manuscript and giving us the chance to submit a revised version.

Please find below (in italics) the point-to-point answers to the reviewers' comments and suggestions.

As suggested by Reviewer #2 we have done a new long-term experiment with R-flurbiprofen in the primary progressive EAE model in C57BL6 mice with a late start of the treatment when the disease had fully developed (day 13). The result (presented now in figure 1D) shows that the scores of mice treated with R-flurbiprofen slowly start to drop and separate from the vehicle treated mice, which maintain high scores up to the end of the observation period. R-flurbiprofen treated mice did not reach baseline but motor functions and body weights were much better than in the controls. In addition, we show that myelin destruction was reduced in these mice.

The changes in the manuscript are highlighted by red lettering.

Reviewer #2 was not convinced of the medical impact of the results. We hope that the demonstration of the therapeutic effects in PP-EAE will ease his/hers doubts. I should like to add that we have received a large grant from the German National Research and Health Ministry (BMBF) to perform a clinical phase 2a study to assess the efficacy of R-flurbiprofen in MS patients. The preclinical results passed the critical evaluation of a board of reviewers.

We thank you and the reviewers for the evaluation of our manuscript and hope that our additional studies and answers pass their critical view and convince reviewer #2 of the medical relevance of the findings. We wish to thank you for handling of our manuscript and look forward to hearing from you.

Regards Irmgard Tegeder

Referee #1 (Comments on Novelty/Model System):

Data are of a high quality and the study may have important medical impact. There are no major issues to address.

Referee #1 (Remarks):

This is an excellent manuscript, in which the effects of R-flurbiprofen as a potential therapeutic agent for multiple sclerosis are explored in EAE models in mouse. Multiple models and clinically

relevant time points for the effectiveness of R-flurbiprofen are measured, and numerous readouts at the behavioral level (nociceptive testing), cellular level (immune cell infiltration, microglial activation etc), and molecular level (microarray analysis) are used to monitor disease progression and potential mechanisms. The authors demonstrate the R-flurbiprofen has strong effects and have gone to some effort to understand the mechanisms of its actions. While they have not been able to discover a single mechanism, they have explored multiple options, and in light of the literature conclude that a combination of mechanisms is probably responsible. In summary, this appears to be an important study and as pointed out by the authors, their data strongly supports further clinical investigations of R-flurbiprofen in multiple sclerosis.

Minor points:

Please explain why the hot plate test and Dynamic Plantar Aesthesiometer tests were used in C57BL6 mice, and Hargreaves test and tail-flick were used in SJL mice.

In the C57BL6 mice we used all tests including heat, cold, mechanical and heat-reflex stimuli. The animals were in good health because the experiments were done before onset of the clinical scores. In the SJL mice we had to restrict the stimuli to reduce the stress of the animals, which is not so much caused by the stimulus per se but more by the time the mouse has to spend outside its home cage in the testing environment. Although the tests were done in the intervals, the mice had already exhibited EAE manifestations as well as some minor symptoms such as ataxia, even in the intervals. We therefore, selected the most robust heat stimuli and used the Hargreaves instead of Hot Plate because it allows for evaluation of left and right hind paws separately and because the Hargreaves is done in the same test cage as the tail flick.

Use "resting" rather than "peaceful" when describing microglial phenotypes.

We have replaced peaceful with resting as suggested.

Referee #2 (Comments on Novelty/Model System):

Technologies and model system applied are adequate. Novelty is high (to my knowledge this is the first time that R-flurbiprofen has been found to be of any therapeutic usefulness rather than being viewed as the inactive isomer of s-flurbiprofen). However, medical impact is medium at the best because compound is very effective in the RR form of MS and much less in the progressive form, which is, indeed, the area with major medical need (the RR form of MS has already some effective medications.

We agree that the biggest clinical challenge is the chronic progressive form of MS. But from the different EAE models one cannot directly infer whether a drug is going to be suitable or less effective in the respective form of MS in humans. Actually, the SJL-EAE (Relapsing remitting) presents a greater challenge to drug treatment, particularly if the treatment is started after the first flare of the disease. Many drugs are tested only in C57BL6 using the preventive treatment regimen. For example, FTY720 (fingolimod) fails in the SJL RR-EAE model even if the treatment is initiated 3 days after immunization and BG12 (dimethylfumarate) fails in most mouse EAE models even in C57BL6 using an early treatment start (unpublished data). It is generally accepted that the chance that a drug will work in humans, increases with the number of EAE models in which it was effective. Hence, we believe that the good efficacy in SJL mice (and we have added late treatment with C57BL6 now as suggested) is an advantage and increases the likelihood that R-flurbiprofen may work in humans.

Referee #2 (Remarks):

This is a potentially interesting study but provides only description of effects without a real interpretation of the findings. Based on data presented, R-flurbiprofen effect in MS is the

consequence of its simultaneous action on several targets, none of which is really decisive. Only the sum of each single contribution gives the final effect.

To become really attractive to the reader, paper needs to be improved. Some comments/suggestions could be the following:

In the current format findings are entirely linked to the peculiar pharmacological profile of R-flurbiprofen. In my view, authors should try to identify if a main molecular target is primarily engaged and drives the efficacy in MS more than the other targets. Then the focus of research (and discussion) should be on this mechanism.

This is undoubtedly a good idea though it is likely, however, to lead to more questions than answers. We have assessed various specific targets of R-flurbiprofen in various models and our conclusion on the mechanistic interrelationships of the different effects is based on this experience with the drug. We present the initial concept of how therapeutic effects may ensue from targeting endocannabinoid transport and metabolism, PPARs and ATP transporters which are all direct targets of R-flurbiprofen in vitro and all play a role in human MS. We could now aim to knock out these factors one by one to see whether the efficacy is altered. However, individual knockout of PPARs, FAAH or MRPs per se will in each case directly affect EAE development and therefore confound interpretation of subsequent drug effects. Such studies will also be very time-consuming and it is questionable whether they would significantly clarify the potential therapeutic value of the drug in human MS. It is also worth pointing out that most of the currently marketed drugs for MS, including fingolimod, BG-12 and interferon-beta, are all thought to act in human MS by multiple mechanisms.

Data on R-Flubiprofen in EAE induced demyelination should be provided. A simple measure of MBP could be sufficient. This will strengthen the concept that myelin sheath destruction is reduced/halted by R-flubiprofen treatment

We have included figures showing that the myelin destruction is reduced in R-flurbiprofen treated mice both in the spinal cord (Figure 7, SJL mice late start of treatment) and in the optic nerves (now Figure 10E, C57BL6 mice on semi-therapeutic treatment).

We have now added in vivo near-infrared imaging (NIRF) of myelin with DBT (3,3'-diethylthiatricarbocyanine iodide) that is a myelin-binding dye allowing for quantitative assessment of non-inflammatory demyelination in the cuprizone model (decrease of DBT binding) or assessment of inflammatory myelin destruction in the EAE model (increase of DBT binding). We have therefore used normal mice and cuprizone-treated mice as controls for the EAE mice treated with vehicle of R-flurbiprofen. For the NIFR imaging, we used C57BL6 mice in which the treatment with R-flurbiprofen was initiated after full development of the disease and imaging was done 39 days after immunization (now Figure 10C and D). The results show that DBT fluorescence of R-flurbiprofen treated mice resembles the normal controls, showing that inflammatory myelin destruction was reduced.

We have also added MBP ELISA data as suggested (now Figure 10 F) again of late-treated C57BL6 mice of the corpus callosum and spinal cord. The results show that vehicle treated mice have lower MBP levels than naïve controls or R-flurbiprofen treated mice whereas MBP in the R-flurbiprofen group does not significantly differ from the naïve mice.

Not clear the extent at which R-flurbiprofen multi-targets are shared by the S-form. Could be helpful to make comparative studies since they might unmask MS related specific targets.

So far, all R-flurbiprofen targets studied are shared with S-flurbiprofen but the potency differs in favor of the R-form by a factor of approximately 10, depending on the target. The major issue with S-flurbiprofen, however, is the inhibition of cyclooxygenase mediated prostaglandin production that is specific for S and does not occur with R (potency of S in vivo is about 100-fold higher). As a consequence, it is not possible to administer S-flurbiprofen for longer than 1-2 weeks in rodents because of its pronounced gastrointestinal toxicity. S-treated rodents rapidly die from GI bleeding. Hence, we could not include S-flurbiprofen as a control drug in most of our EAE experiments.

Not clear the contribution of the microarray study. As expected several genes were found up-regulated, others were found down-regulated and the expression of others did not change. Authors should better discuss the impact of these findings (favorite option) or, if not impact, withdraw them.

The microarray study was done using score-matched pairs. Therefore, the differences in the gene expression reveal R-flurbiprofen-specific patterns and groups of regulated genes, thereby helping to understand its effects. We therefore, think that the gene expression pattern is relevant to the therapeutic effects observed and supports the behavioral studies. We further carried out an overrepresentation analysis (ORA) to evaluate prominent patterns (Suppl. Table 9 and the hierarchy is shown in suppl. Figure 1). The ORA results are discussed on page 11. The ontology annotations of the genes that differed most strongly between R-flurbiprofen and vehicle, pointed towards "cell adhesion" and "cell surface receptor-linked signaling" as potential R-flurbiprofen regulated processes, whereas metabolic processes (i.e. nucleic acid and protein synthesis and metabolism) were underrepresented.

Authors found that R-flubiprofen can prevent/reduce EAE development in C57 mice (model of progressive MS). Wondering if drug can also reverse established symptoms in diseased animals (curative protocol). This would provide much greater medical impact.

As requested by the reviewer, we have further assessed the effects of R-flurbiprofen in C57BL6 mice in which the treatment was started only after full development of the disease. The animals were allocated to R-flurbiprofen 5 mg/kg/d orally or vehicle as score-matched pairs after reaching peak EAE scores (day 13 after immunization). R-flurbiprofen and vehicle were administered with drug- or vehicle-soaked sweet cornflakes to which the mice were "habituated" before onset of the clinical scores so that they were accustomed to eat these goodies and received sufficient drug, fluid and calories during the disease. The results of this study are now presented as Figure 1D (the subpanels of Figure 1 have been rearranged). They show that the scores in R-flurbiprofen treated mice slowly drop whereas those in vehicle treated mice stay around a score of 2.5-3. The differences become significant after about 10 days of treatment. The rm-ANOVA is highly significant (P < 0.001) and the body weight of R-flurbiprofen treated mice was significantly higher at the end of the treatment period. So far, few drugs have been shown previously to provide such a late therapeutic effect in C57BL6 mice, but were mostly started on day 3 to achieve a comparable therapeutic effect e.g. BG12 {Linker, 2011 #15800}.

2nd Editorial Decision 21 August 2014

Thank you for the submission of your revised manuscript to EMBO Molecular Medicine. We have now received the enclosed report from the referee who was asked to re-assess it. As you will see this reviewer is now supportive and I am pleased to inform you that we will be able to accept your manuscript pending following final editorial amendments.

Please submit your revised manuscript within two weeks. I look forward to seeing a revised form of your manuscript as soon as possible.

***** Reviewer's comments *****

Referee #2 (Comments on Novelty/Model System):

The paper is now in a much better shape than it was before and is potentially very interesting. In the present version a proper point by reply to my previous comments has been included. In particular I am very happy of the demonstration that r-flurbiprofen is effective also when given once disease is initiated. This finding significantly increases the medical value of the findings.

Referee #2 (Remarks):

Paper is now of more impact than the previous version. The reported finding that r-flurbiprofen is still effective after disease (EAE) induction increases significantly the medical impact of the study. The mechanism of action remains unclear. This lack can be still perceived as an important weakness.
