# Supplementary figures and images for: R-flurbiprofen attenuates experimental autoimmune encephalomyelitis in mice
Source: EMBO Mol Med. 2014 Sep 30;6(11):1398–422. doi: 10.15252/emmm.201404168 (PMC4237468; doi:10.15252/emmm.201404168)

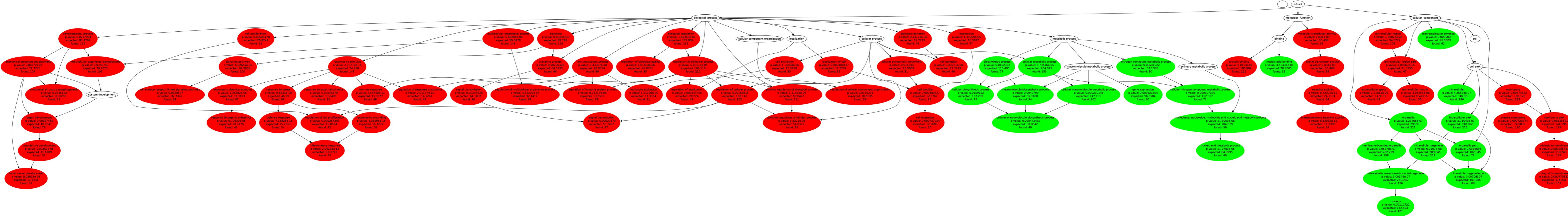

Supplement: Supplementary file 2 [file emmm0006-1398-sd2.pdf]
