# Supplementary material for: R-flurbiprofen attenuates experimental autoimmune encephalomyelitis in mice
Source: EMBO Mol Med. 2014 Sep 30;6(11):1398–422. doi: 10.15252/emmm.201404168 (PMC4237468; doi:10.15252/emmm.201404168)
Supplement: Supplementary file 3 [file emmm0006-1398-sd3.pdf]

Contents

Suppl. Table 1a 2

Antibodies used for Flow cytometry 2

Suppl. Table 1b 2

Antibodies used for immunofluorescence and Western blots 2

Suppl. Table 2 3

Microglia and macrophages in the spinal cord in C57BL6 mice with preventive R-flurbiprofen treatment versus vehicle 3

Suppl. Table 3 3

Microglia and macrophages in the spinal cord in C57BL6 mice with semi-therapeutic R-flurbiprofen 3

Suppl. Table 4 4

Spinal cord T-cells and macrophages of C57BL6 mice with EAE after receiving a β-actin EGFP bone marrow transplant: treatment with R-flurbiprofen versus vehicle 4

Suppl. Table 5 4

Splenic T-cells of C57BL6 mice with EAE after receiving a β-actin EGFP bone marrow transplant: treatment with R-flurbiprofen versus vehicle 4

Suppl. Table 6 5

Splenic T-cells of C57BL6/J mice with-semi therapeutic R-flurbiprofen treatment versus vehicle 5

Suppl. Table 7 5

Splenic T-cells of SJL mice with EAE and semi-therapeutic treatment with R-flurbiprofen versus vehicle 5

Suppl. Table 8 6

Top 100 genes which were less upregulated in EAE mice on R-flurbiprofen treatment as compared to vehicle treated mice 6

Suppl. Table 9 9

Gene ontology over-representation analysis for differentially regulated genes 9

Legend to Suppl. Figure 1 10

Suppl. Table 1a

Antibodies used for Flow cytometry

Antibody	Conjugate	Company	Concentration	Marker for
CD45	PerCPeFluor710	BD	0.2 mg/ml	Immune cells
CD45	VioBlue	Miltenyi Biotec	0.2 mg/ml	Immune cells
CD3e	PacificBlue	BD	0.2 mg/ml	T-cells
CD3e	APC-Cy7	Biolegend	0.2 mg/ml	T-cells
CD4	PE	BD	0.2 mg/ml	T-helper cells
CD25	AlexaFluor488	eBioscience	0.5 mg/ml	Regulatory T-cells
CD25	APC	eBioscience	0.2 mg/ml	Regulatory T-cells
CD8	FITC	eBioscience	0.5 mg/ml	T-suppressor cells
CD8	PerCPeFluor710	eBioscience	0.2 mg/ml	T-suppressor cells
CD206	FITC	Biolegend	0.5 mg/ml	M2-like Macrophages
CD11b	APC	Miltenyi Biotec	0.2 mg/ml	Macrophages / microglia
CD11b	eFluor450	eBioscience	0.2 mg/ml	Macrophages / microglia
CD11c	eFlour450	eBioscience	0.2 mg/ml	Dendritic cells / monocytes
MHCII	PE	eBioscience	0.2 mg/ml	Antigen presenting cells
MHCII	PerCPeFluor710	eBioscience	0.2 mg/ml	Antigen presenting cells
CD183 (CXCR3)	APC	eBioscience	0.2 mg/ml	Resident microglia / monocytes
CD36 (Scavenger receptor class B)	PE	eBioscience	0.2 mg/ml	Monocytes/Macrophages
CD152/CTLA4	APC	eBioscience	0.2 mg/ml	Inhibitory T-helper cells
Foxp3	eFluor450	eBioscience	0.2 mg/ml	Regulatory T-cells
IFNγ	AlexaFluor488	eBioscience	0.5 mg/ml	Pro-inflammatory, T-helper cells
IL10	APC	eBioscience	0.2 mg/ml	Anti-inflammatory, Th2-cells
IL17A	PE	eBioscience	0.2 mg/ml	Pro-inflammatory, Th17-cells

Suppl. Table 1b

Antibodies used for immunofluorescence and Western blots

Antibody	Host	Conjugate	Company	Concentration	Marker for
Fluoromyelin		Texas Red 558/654 nm	Life technologies		Myelin
F4/80	rat	Unconjugated	eBioscience	0.5 mg/ml	Myeloid cells
CD11b	rat	Unconjugated	Serotec	1 mg/ml	Macrophages / microglia
GFAP	rabbit	Unconjugated	Sigma		Astrocytes
Iba1	rabbit	Unconjugated	Santa Cruz	0.2 mg/ml	Macrophages / microglia
MBP	mouse	Unconjugated	Covance	1 mg/ml	Myelin
Arginase 1	goat	Unconjugated	Santa Cruz	0.2 mg/ml	M2-like macrophages / microglia
NF200	rabbit	Unconjugated	Sigma		Neurofilament
NeuN	mouse	Unconjugated	Millipore	1 mg/ml	Neuronal nuclei
NIMP-R14	rat	Unconjugated			Neutrophils

Suppl. Table 2

Microglia and macrophages in the spinal cord in C57BL6 mice with preventive R-flurbiprofen treatment versus vehicle

n=3, mean ± SD	Naive			Vehicle			R-Flurbi			Statistics P-values	
Population	% of Parent		SD	% of Parent		SD	% of Parent		SD	Naive vs Veh	Veh vs R-Flurbi
CD45+	9.0	±	1.8	13.8	±	4.0	10.4	±	0.3	ns	ns
CD11b-high_CD206(-)	1.1	±	0.1	15.2	±	10.1	1.5	±	0.1	0.0093	0.0126
CD11b-high_CD206+	0.3	±	0.0	3.0	±	2.8	0.4	±	0.2	ns	ns
CD11b-low_CD206(-)	76.3	±	2.2	53.9	±	16.9	76.7	±	5.8	0.0001	0.0001
CD11b-low_MHC(-)	64.1	±	2.7	50.7	±	13.3	65.0	±	9.0	0.0171	0.0079
CD11b-low_MHC+	2.0	±	0.7	17.8	±	12.9	3.6	±	1.9	0.0018	0.0082
CD11b-low_CXCR3-low	71.2	±	1.7	52.8	±	12.9	70.0	±	5.4	0.0002	0.0005
CD11b-high_CXCR3-low	1.7	±	0.1	16.5	±	10.8	2.0	±	0.1	0.005	0.0062
CD11b.low_CXCR3-high	2.1	±	1.0	2.4	±	0.9	2.5	±	0.7	ns	ns
CD11b.high_CXCR3-high	0.2	±	0.0	4.3	±	2.2	0.7	±	0.3	ns	ns

C57BL6/J mice, myeloid cells in the spinal cord, preparation during the flare of the disease, day 20. R-flurbiprofen treatment 10 mg/kg/d in the drinking water, start 3 d after immunization (preventive).

Statistics: FACS data were analyzed by two-way ANOVA using 'population' as within and 'treatment' as between subject factors, followed by post hoc analyses using a Bonferroni correction for alpha.

Suppl. Table 3

Microglia and macrophages in the spinal cord in C57BL6 mice with semi-therapeutic R-flurbiprofen

	Naive			Vehicle			R-Flurbi			Statistics P-values
n=3 mean SD	% of Parent		SD	% of Parent		SD	% of Parent		SD	Veh vs R-Flurbi
CD45	4,20	±	0,82	7,54	±	0,88	8,06	±	1,71	ns
CD11b and/or CXCR3	5,23	±	1,37	8,56	±	3,71	7,44	±	0,87	ns
CD11b	5,40	±	0,54	13,00	±	1,30	7,30	±	0,73	ns
CD11b_MHC-II(-) CXCR3+	53,50	±	6,30	53,00	±	5,30	59,00	±	4,90	ns
CD11b_MHC-II+ CXCR3+	4,75	±	0,47	15,00	±	1,52	3,00	±	0,30	0.0013
CD11b_MHC-II+ CXCR3(-)	0,60	±	0,06	1,70	±	0,14	1,40	±	0,17	ns
CD11b_MHC-II(-) CXCR3(-)	42,00	±	3,20	30,00	±	3,20	37,00	±	3,70	ns
CD11b_CD36(-) CD206+	3,80	±	0,37	5,90	±	1,59	3,10	±	1,31	ns
CD11b_CD36+ CD206+	13,00	±	3,30	0,70	±	0,17	4,10	±	0,41	ns
CD11b_CD36+ CD206(-)	56,00	±	4,60	0,10	±	0,01	65,00	±	6,50	0.000007
CD11b_CD36(-) CD206(-)	28,00	±	2,83	93,00	±	15,30	28,00	±	3,80	0.000013

C57BL6/J mice, myeloid cells in the spinal cord, preparation during the flare of the disease, day 20. R-flurbiprofen treatment 10 mg/kg/d in drinking water, 4 days before onset of clinical scores (semi-therapeutic).

Statistics: FACS data were analyzed by two-way ANOVA using 'population' as within and 'treatment' as between subject factors, followed by post hoc analyses using a Bonferroni correction for alpha.

Suppl. Table 4

Spinal cord T-cells and macrophages of C57BL6 mice with EAE after receiving a β-actin EGFP bone marrow transplant: treatment with R-flurbiprofen versus vehicle

n=4, mean ±SD	Naive			Vehicle			R-Flurbi			Statistics P-values	
Population	Counts			Counts			Counts			N vs Veh	Veh vs R-Flurbi
β-actin-low.CD4+	252.5	±	37.5	749.5	±	172.6	471.3	±	180.7	0.0337	ns
β-actin-high.CD4+	17.0	±	1.2	538.0	±	86.1	301.0	±	253.5	0.0262	ns
β-actin-low.CD11b+	499.5	±	131.1	2150.0	±	462.2	1186.3	±	591.4	0.0001	0.0019
β-actin-high.CD11b+	88.5	±	15.6	387.5	±	58.9	252.8	±	122.0	ns	ns
β-actin+CD25+	1702.5	±	47.9	2675.8	±	1196.4	1914.8	±	369.8	0.0016	0.0379
CD11b-low.CD25+	431.0	±	50.8	841.5	±	113.1	704.8	±	160.0	ns	ns
CD11-high.CD25+	17.5	±	5.2	806.8	±	342.4	528.5	±	391.6	0.0256	ns
CD4+CD25low	100.0	±	12.7	820.3	±	345.9	519.0	±	344.2	0.052	ns
CD4+CD25high	16.5	±	0.6	85.0	±	35.2	54.0	±	34.5	ns	ns

C57BL6/J mice after bone marrow transplantation from β-actin-EGFP donor mice. Immunization 3 weeks after irradiation. R-flurbiprofen 10 mg/kg/d in the drinking water from day 3 after immunization. Preparation of spinal cords during the flare of the disease, day 16.

Statistics: FACS data were analyzed by two-way ANOVA using 'population' as within and 'treatment' as between subject factors, followed by post hoc analyses using a Bonferroni correction for alpha.

Suppl. Table 5

Splenic T-cells of C57BL6 mice with EAE after receiving a β-actin EGFP bone marrow transplant: treatment with R-flurbiprofen versus vehicle

n=4, mean ± SD	Vehicle			R-flurbiprofen			Statistics
Population	% of Parent		SD	% of Parent		SD	P-values
β-actin(-)	39.3	±	2.5	52.7	±	6.3	0.0151
β-actin(-)CD4+	11.4	±	1.7	6.4	±	1.3	ns
β-actin(-)_CD4+_CD25(-)FoxP3+	6.1	±	1.9	18.2	±	3.5	0.0320
β-actin(-)_CD4+_CD25+FoxP3+	3.5	±	0.9	14.7	±	4.6	0.0495
β-actin+	37.7	±	3.1	29.3	±	6.5	ns
β-actin+CD4+	44.7	±	3.4	59.4	±	4.8	0.0066
β-actin+CD4+_CD25(-)FoxP3+	12.6	±	6.6	30.1	±	4.1	0.0008
β-actin+CD4+_CD25+FoxP3+	6.5	±	3.4	36.9	±	18.4	0.0001
β-actin+_CD4+CD25+	2.3	±	0.6	12.9	±	9.3	ns
CD4+	25.5	±	1.9	19.8	±	5.3	ns
CD4+_CD25+FoxP3+	0.1	±	0.1	7.0	±	7.1	ns

C57BL6/J mice after bone marrow transplantation from β-actin-EGFP donor mice. Immunization 3 weeks after irradiation. R-flurbiprofen 10 mg/kg/d in the drinking water from day 3 after immunization. Preparation of splenocytes during the flare of the disease, day 16.

Statistics: FACS data were analyzed by two-way ANOVA using 'population' as within and 'treatment' as between subject factors, followed by post hoc analyses using a Bonferroni correction for alpha.

Suppl. Table 6

Splenic T-cells of C57BL6/J mice with-semi therapeutic R-flurbiprofen treatment versus vehicle

n=3, mean ± SD	Naive			Vehicle			R-flurbiprofen			Statistics
Population	% Parent		SD	% Parent		SD	% Parent		SD	Veh vs. R-Flurbi
CD4+	24.9	±	4.3	42.2	±	5.4	43.7	±	6.9	ns
CD4+FoxP3+	5.9	±	1.7	5.04	±	0.7	5.06	±	1.4	ns
CD4+FoxP3+IL10+	11.7	±	2.1	14.7	±	2.2	47.1	±	8.2	0.0001
CD4+IL10+	1.0	±	0.2	0.84	±	0.3	2.55	±	0.4	0.04
IL-10+	9.0	±	1.6	4.98	±	1.0	6.18	±	1.5	ns
FoxP3(-)IL10+	3.8	±	0.2	5.75	±	1.2	1.67	±	1.1	ns
FoxP3+IL10+	10.2	±	1.8	5.69	±	1.0	16.3	±	1.8	0.004
FoxP3+IL10(-)	17.1	±	2.4	11.5	±	2.0	9.76	±	1.7	ns
FoxP3(-)IL10(-)	69.0	±	12.0	77.1	±	5.1	72.2	±	4.6	ns

C57BL6/J mice during the flare, R-flurbiprofen 10 mg/kg/d in the drinking water, start of treatment 4 days before onset of scores (semi-therapeutic). Splenocytes were restimulated with 50 ng/ml PMA + 500 ng/ml ionomycin for 2h, 37°C. Cytokine release block with brefeldin A 10 µg/ml for 2h, 37°C.

Statistics: FACS data were analyzed by two-way ANOVA using 'population' as within and 'treatment' as between subject factors, followed by post hoc analyses using a Bonferroni correction for alpha.

Suppl. Table 7

Splenic T-cells of SJL mice with EAE and semi-therapeutic treatment with R-flurbiprofen versus vehicle

n = 10, mean ± SD	Vehicle			R-flurbiprofen			
Population	% of Parent		SD	% of Parent		SD	Statistics
CD3+	31.01	±	3.99	21.37	±	5.39	0.0015
CD3+CD8+	27.53	±	3.35	21.78	±	1.30	ns
CD3+CD8+CD152+	18.28	±	11.99	32.99	±	9.80	0.0003
CD3+CD8+CD152(-)	74.36	±	12.66	57.77	±	9.39	0.0040
CD3+CD4+	62.65	±	2.66	66.99	±	1.73	ns
CD3+CD4+CD152+	7.77	±	7.86	17.44	±	7.94	0.0033
CD3+CD4+CD152(-)	87.97	±	8.45	77.77	±	7.59	0.0032
CD8+	9.59	±	1.96	5.45	±	1.25	0.0104
CD8+CD25+	2.25	±	0.79	3.63	±	1.44	0.0013
CD4+	21.91	±	2.58	16.99	±	3.98	0.0281
CD4+CD25+	2.546	±	0.68	3.55	±	0.63	0.0267
CD4+CD152+	10.712	±	8.40	20.30	±	8.05	0.0073
CD4+CD152+CD25+	0.781	±	0.60	1.14	±	0.39	ns

FACS analysis of T-cells in the spleen of SJL mice during the 1st EAE flare. R-flurbiprofen 5 mg/kg/d in the drinking water, start 5d after immunization (semi-therapeutic). CD152 = CTLA4 (Cytotoxic T-Lymphocyte Antigen 4) is a marker for T-cells that down-regulate an activated immune system.

Statistics: FACS data were analyzed by two-way ANOVA using 'population' as within and 'treatment' as between subject factors, followed by post hoc analyses using a Bonferroni correction for alpha.

Suppl. Table 8

Top 100 genes which were less upregulated in EAE mice on R-flurbiprofen treatment as compared to vehicle treated mice

		Gene expression mean intensity (AU)					
Accession	Symbol	CFA-Vehicle	CFA-R-Flurbi	EAE-Vehicle	EAE-R-Flurbi	Protein product	Definition
NM_178185.1	Hist1h2ao	2743.47	2249.86	9214.45	3846.86	NM_178185.1	Mus musculus histone cluster 1, H2ao (Hist1h2ao), mRNA.
NM_016740.3	S100a11	2702.15	2146.56	15077.89	6192.28	NP_058020.1	Mus musculus S100 calcium binding protein A11 (calgizzarin) (S100a11), mRNA.
NM_007542.3	Bgn	2509.33	2340.65	6863.45	2862.38	NP_031568.2	Mus musculus biglycan (Bgn), mRNA.
NM_017372.3	Lyz2	1826.68	998.44	17082.36	6087.44	NP_059068.1	Mus musculus lysozyme 2 (Lyz2), mRNA.
NM_008175.3	Grn	1462.04	1347.53	11592.11	4133.82	NP_032201.1	Mus musculus granulin (Grn), mRNA.
NM_009663.1	Alox5ap	1354.91	772.07	8300.58	3184.84	NP_033793.1	Mus musculus arachidonate 5-lipoxygenase activating protein (Alox5ap), mRNA.
NM_007792.3	Csrp2	1202.46	1336.73	2453.16	1029.27	NM_007792.3	Mus musculus cysteine and glycine-rich protein 2 (Csrp2), mRNA.
NM_010517.3	Igfbp4	1119.60	1067.15	3339.25	1239.43	NP_034647.1	Mus musculus insulin-like growth factor binding protein 4 (Igfbp4), mRNA.
NM_010705.2	Lgals3	1091.12	764.05	15570.30	6174.99	NP_034835.1	Mus musculus lectin, galactose binding, soluble 3 (Lgals3), mRNA.
NM_008183.3	Gstm2	1004.19	1181.14	2222.57	904.10	NP_032209.1	Mus musculus glutathione S-transferase, mu 2 (Gstm2), mRNA.
NM_001034097.1	Tnfsf12-tnfsf13	902.41	1073.75	2954.69	1124.02	NP_001029269.1	Mus musculus tumor necrosis factor (ligand) superfamily, member 12-member 13 (Tnfsf12-tnfsf13), transcript variant 1, mRNA.
NM_008908.1	Ppic	855.36	980.36	3327.58	1312.79	NP_032934.1	Mus musculus peptidylprolyl isomerase C (Ppic), mRNA.
NM_008161.2	Gpx3	838.87	713.48	4045.89	1246.00	NP_032187.2	Mus musculus glutathione peroxidase 3 (Gpx3), transcript variant 2, mRNA.
NM_008039.2	Fpr2	819.55	406.84	6675.74	2594.26	NP_032065.1	Mus musculus formyl peptide receptor 2 (Fpr2), mRNA.
NM_007426.3	Angpt2	808.02	855.42	1873.18	746.43	NP_031452.2	Mus musculus angiopoietin 2 (Angpt2), mRNA.
NM_017372.2	Lyzs	737.21	510.56	6403.37	2554.39	NP_059068.1	Mus musculus lysozyme (Lyzs), mRNA.
NM_133198.1	Pygl	728.19	523.36	2834.96	1141.12	NP_573461.1	Mus musculus liver glycogen phosphorylase (Pygl), mRNA.
NM_013739.2	Dok3	697.05	728.38	2364.50	989.56	NM_013739.2	Mus musculus docking protein 3 (Dok3), mRNA.
NM_026436.3	Tmem86a	654.39	686.67	2055.27	832.37	NP_080712.1	Mus musculus transmembrane protein 86A (Tmem86a), mRNA.
NM_015734.1	Col5a1	640.57	831.39	2120.37	688.95	NP_056549.1	Mus musculus procollagen, type V, alpha 1 (Col5a1), mRNA.
NM_011414.2	Slpi	622.87	347.18	2128.69	588.62	NP_035544.1	Mus musculus secretory leukocyte peptidase inhibitor (Slpi), mRNA.
NM_145126.1	Chi3l4	606.66	375.22	8332.35	1552.28	NP_660108.1	Mus musculus chitinase 3-like 4 (Chi3l4), mRNA.
NM_010740.3	Cd93	604.54	487.38	2093.96	649.20	NP_034870.1	Mus musculus CD93 antigen (Cd93), mRNA.
NM_026376.3	Plxnd1	573.89	583.24	2172.55	759.08	NP_080652.2	Mus musculus plexin D1 (Plxnd1), mRNA.
NM_010728.1	Lox	562.84	636.16	2240.46	793.56	NP_034858.1	Mus musculus lysyl oxidase (Lox), mRNA.
NM_007681.2	Cenpa	559.14	511.07	2855.60	1064.95	NP_031707.1	Mus musculus centromere protein A (Cenpa), mRNA.
NM_175659.1	Hist1h2ah	539.95	470.67	2437.46	993.62	NP_783590.1	Mus musculus histone cluster 1, H2ah (Hist1h2ah), mRNA.
NM_011990.2	Slc7a11	521.67	439.93	2050.53	714.44	NP_036120.1	Mus musculus solute carrier family 7 (cationic amino acid transporter, y+ system), member 11 (Slc7a11), mRNA.
NM_010729.2	Loxl1	488.54	444.13	2002.56	749.22	NP_034859.2	Mus musculus lysyl oxidase-like 1 (Loxl1), mRNA.
NM_028784.2	F13a1	487.60	334.74	2001.46	440.80	NP_083060.2	Mus musculus coagulation factor XIII, A1 subunit (F13a1), mRNA.
NM_009841.3	Cd14	477.04	247.93	4806.94	1375.82	NP_033971.1	Mus musculus CD14 antigen (Cd14), mRNA.
NM_009349.3	Inmt	464.70	461.40	1991.23	220.69	NP_033375.1	Mus musculus indolethylamine N-methyltransferase (Inmt), mRNA.
NM_007392.2	Acta2	460.04	423.57	1197.17	422.44	NP_031418.1	Mus musculus actin, alpha 2, smooth muscle, aorta (Acta2), mRNA.
NM_009369.3	Tgfbi	459.64	410.74	5372.81	1977.08	NP_033395.1	Mus musculus transforming growth factor, beta induced (Tgfbi), mRNA.
NM_011662.2	Tyrobp	459.22	341.45	3447.84	1467.13	NP_035792.1	Mus musculus TYRO protein tyrosine kinase binding protein (Tyrobp), mRNA.
NM_001008702.1	Dab2	443.56	427.31	3347.97	1011.92	NP_001008702.1	Mus musculus disabled homolog 2 (Drosophila) (Dab2), transcript variant 2, mRNA.
NM_031397.2	Bicc1	422.42	447.19	1097.06	421.26	NP_113574.1	Mus musculus bicaudal C homolog 1 (Drosophila) (Bicc1), mRNA.
NM_007899.1	Ecm1	394.19	393.80	3664.06	749.21	NP_031925.1	Mus musculus extracellular matrix protein 1 (Ecm1), mRNA.
NM_008342.2	Igfbp2	388.20	390.06	925.55	333.19	NP_032368.2	Mus musculus insulin-like growth factor binding protein 2 (Igfbp2), mRNA.

XM_001480162.1	Fer1l3	380.44	323.48	2296.83	899.62	XP_001480212.1	PREDICTED: Mus musculus fer-1-like 3, myoferlin (C. elegans), transcript variant 1 (Fer1l3), mRNA.
NM_028903.1	Scara5	366.80	476.22	1109.87	393.50	NP_083179.1	Mus musculus scavenger receptor class A, member 5 (putative) (Scara5), mRNA.
NM_053110.3	Gpnmb	364.32	434.53	8533.03	811.09	NP_444340.2	Mus musculus glycoprotein (transmembrane) nmb (Gpnmb), mRNA.
NM_008549.1	Man2a1	358.61	365.15	1935.25	817.96	NP_032575.1	Mus musculus mannosidase 2, alpha 1 (Man2a1), mRNA.
NM_011347.1	Selp	344.60	169.07	2400.38	907.53	NP_035477.1	Mus musculus selectin, platelet (Selp), mRNA.
NM_175097.3	Prickle3	338.73	357.67	1114.61	478.80	NP_780306.1	Mus musculus prickle homolog 3 (Drosophila) (Prickle3), mRNA.
NM_010233.1	Fn1	331.58	289.52	3079.86	891.89	NP_034363.1	Mus musculus fibronectin 1 (Fn1), mRNA.
NM_201641.2	Ugt1a10	322.79	346.78	1590.00	618.45	NP_964003.2	Mus musculus UDP glycosyltransferase 1 family, polypeptide A10 (Ugt1a10), mRNA.
NM_031254.2	Trem2	322.71	318.50	1842.32	586.81	NP_112544.1	Mus musculus triggering receptor expressed on myeloid cells 2 (Trem2), mRNA.
NM_023132.2	Renbp	318.20	357.56	1402.64	546.87	NP_075621.2	Mus musculus renin binding protein (Renbp), mRNA.
NM_145150.1	Prc1	316.08	293.18	1825.85	702.08	NP_660132.1	Mus musculus protein regulator of cytokinesis 1 (Prc1), mRNA.
NM_008380.1	Inhba	307.19	271.81	1426.82	613.32	NP_032406.1	Mus musculus inhibin beta-A (Inhba), mRNA.
NM_007925.3	Eln	303.79	274.66	914.59	364.82	NP_031951.2	Mus musculus elastin (Eln), mRNA.
NM_001081437.1	Fbln2	286.44	318.64	2024.86	449.03	NP_001074906.1	Mus musculus fibulin 2 (Fbln2), transcript variant 2, mRNA.
NM_173371.3	H6pd	285.62	290.61	1299.77	551.44	NP_775547.2	Mus musculus hexose-6-phosphate dehydrogenase (glucose 1-dehydrogenase) (H6pd), mRNA.
NM_011338.2	Ccl9	285.18	241.24	6958.17	2115.19	NP_035468.1	Mus musculus chemokine (C-C motif) ligand 9 (Ccl9), mRNA.
NM_009994.1	Cyp1b1	282.51	289.23	1475.65	610.05	NP_034124.1	Mus musculus cytochrome P450, family 1, subfamily b, polypeptide 1 (Cyp1b1), mRNA.
NM_009994.1	Cyp1b1	282.51	289.23	1475.65	610.05	NP_034124.1	Mus musculus cytochrome P450, family 1, subfamily b, polypeptide 1 (Cyp1b1), mRNA.
NM_173442.2	Gcnt1	277.71	252.02	800.56	329.16	NP_775618.1	Mus musculus glucosaminyl (N-acetyl) transferase 1, core 2 (Gcnt1), mRNA.
NM_009853.1	Cd68	270.51	242.26	3534.51	1019.87	NP_033983.1	Mus musculus CD68 antigen (Cd68), mRNA.
NM_007782.1	Csf3r	261.12	227.70	1216.46	466.28	NP_031808.1	Mus musculus colony stimulating factor 3 receptor (granulocyte) (Csf3r), mRNA.
NM_008489.2	Lbp	254.43	245.47	945.85	320.85	NP_032515.2	Mus musculus lipopolysaccharide binding protein (Lbp), mRNA.
NM_009892.1	Chi3l3	244.62	168.68	2372.97	343.58	NP_034022.1	Mus musculus chitinase 3-like 3 (Chi3l3), mRNA.
NM_007472.2	Aqp1	243.31	281.51	1842.63	485.22	NP_031498.1	Mus musculus aquaporin 1 (Aqp1), mRNA.
NM_020568.2	S3-12	241.41	256.76	735.05	271.70	NP_065593.1	Mus musculus plasma membrane associated protein, S3-12 (S3-12), mRNA.
NM_007408.3	Adfp	234.24	239.10	1099.28	380.58	NP_031434.3	Mus musculus adipose differentiation related protein (Adfp), mRNA.
NM_007739.2	Col8a1	223.58	219.49	946.27	360.09	NP_031765.2	Mus musculus collagen, type VIII, alpha 1 (Col8a1), mRNA.
NM_133754.3	Fblim1	221.20	202.79	1146.81	415.22	NP_598515.3	Mus musculus filamin binding LIM protein 1 (Fblim1), mRNA.
NM_026414.2	Asprv1	213.57	161.43	1076.27	244.48	NP_080690.2	Mus musculus aspartic peptidase, retroviral-like 1 (Asprv1), mRNA.
NM_138672.2	Stab1	209.38	221.48	832.33	309.40	NP_619613.2	Mus musculus stabilin 1 (Stab1), mRNA.
NM_009909.3	Il8rb	205.90	153.30	533.43	220.27	NP_034039.1	Mus musculus interleukin 8 receptor, beta (Il8rb), mRNA.
NM_010819.3	Clec4d	197.09	165.69	3459.59	640.31	NP_034949.2	Mus musculus C-type lectin domain family 4, member d (Clec4d), mRNA.
NM_205820.1	Tlr13	196.49	169.62	1097.99	422.13	NP_991389.1	Mus musculus toll-like receptor 13 (Tlr13), mRNA.
NM_010924.1	Nnmt	194.36	186.49	834.97	350.37	NP_035054.1	Mus musculus nicotinamide N-methyltransferase (Nnmt), mRNA.
NM_177337.3	Arl11	192.73	180.51	1522.75	510.94	NP_796311.2	Mus musculus ADP-ribosylation factor-like 11 (Arl11), mRNA.
NM_009695.2	Apoc2	192.71	179.84	1392.32	319.98	NP_033825.1	Mus musculus apolipoprotein C-II (Apoc2), mRNA.
NM_018827.2	Crlf1	189.75	197.72	1156.90	318.97	NP_061297.1	Mus musculus cytokine receptor-like factor 1 (Crlf1), mRNA.
NM_013489.1	Cd84	187.85	185.31	1493.00	563.05	NP_038517.1	Mus musculus CD84 antigen (Cd84), mRNA.
NM_023125.2	Kng1	186.51	186.23	1368.65	291.24	NP_075614.1	Mus musculus kininogen 1 (Kng1), mRNA.
NM_021293.2	Cd33	176.86	163.78	1068.33	354.08	NP_067268.1	Mus musculus CD33 antigen (Cd33), mRNA.
NM_027836.5	Ms4a7	175.52	174.41	1149.98	361.79	NP_082112.3	Mus musculus membrane-spanning 4-domains, subfamily A, member 7 (Ms4a7), transcript variant 1, mRNA.
NM_010555.4	Il1r2	175.31	153.61	1181.74	346.23	NP_034685.1	Mus musculus interleukin 1 receptor, type II (Il1r2), mRNA.
NM_011113.3	Plaur	169.48	174.92	864.11	348.24	NP_035243.1	Mus musculus plasminogen activator, urokinase receptor (Plaur), mRNA.
NM_001077189.1	Fcgr2b	165.97	141.68	1652.81	642.12	NP_001070657.1	Mus musculus Fc receptor, IgG, low affinity IIb (Fcgr2b), transcript variant 1, mRNA.
NM_026835.2	Ms4a6d	155.70	146.26	1995.78	855.31	NP_081111.1	Mus musculus membrane-spanning 4-domains, subfamily A, member 6D (Ms4a6d), mRNA.
NM_031167.3	Il1rn	155.57	151.40	2091.58	765.83	NP_112444.1	Mus musculus interleukin 1 receptor antagonist (Il1rn), transcript variant 1, mRNA.
NM_001042489.1	Hvcn1	151.74	163.25	773.52	324.98	NP_001035954.1	Mus musculus hydrogen voltage-gated channel 1 (Hvcn1), transcript variant 1, mRNA.
NM_008372.3	Il7r	148.13	161.79	1120.28	350.88	NP_032398.3	Mus musculus interleukin 7 receptor (Il7r), mRNA.
NM_145634.2	Cd300lf	147.92	133.72	660.70	284.41	NP_663609.2	Mus musculus CD300 antigen like family member F (Cd300lf), mRNA.

NM_019984.2	Tgm1	147.50	151.32	2867.45	956.28	NP_064368.2	Mus musculus transglutaminase 1, K polypeptide (Tgm1), mRNA.
NM_010172.2	F7	147.03	142.33	620.18	175.40	NP_034302.1	Mus musculus coagulation factor VII (F7), mRNA.
NM_176913	Dpep2	147.00	137.37	776.15	229.23	NP_795887.2	Mus musculus dipeptidase 2 (Dpep2), mRNA.
NM_007482	Arg1	141.87	164.49	11710.46	2856.73	NP_031508.1	Mus musculus arginase 1, liver (Arg1), mRNA.
NM_016669.1	Crym	139.10	146.47	503.50	193.16	NP_057878.1	Mus musculus crystallin, mu (Crym), mRNA.
NM_008134.2	Glycam1	138.08	141.95	682.03	219.17	NP_032160.1	Mus musculus glycosylation dependent cell adhesion molecule 1 (Glycam1), mRNA.
NM_022430.2	Ms4a8a	137.73	131.16	974.42	307.28	NP_071875.2	Mus musculus membrane-spanning 4-domains, subfamily A, member 8A (Ms4a8a), mRNA.
NM_008605.3	Mmp12	136.00	135.44	607.44	217.01	NP_032631.3	Mus musculus matrix metallopeptidase 12 (Mmp12), mRNA.
NM_009705.1	Arg2	133.31	135.41	513.41	206.02	NP_033835.1	Mus musculus arginase type II (Arg2), mRNA.
NM_030701.1	Gpr109a	130.67	125.44	1761.86	748.84	NP_109626.1	Mus musculus G protein-coupled receptor 109A (Gpr109a), mRNA.

Suppl. Table 9

Gene ontology over-representation analysis for differentially regulated genes

Functional area (GO Category)	GoTerm ID	Remarkability [%]
**Over-represented**		
**response to stimulus**	GO:0050896	82
response to stress	GO:0006950	64
response to wounding	GO:0009611	35
regulation of response to stimulus	GO:0048583	23
inflammatory response	GO:0006954	15
response to organic substance	GO:0010033	11
**immune system process**	GO:0002376	50
immune response	GO:0006955	27
inflammatory response	GO:0006954	15
leukocyte activation	GO:0045321	2
**multicellular organismal development**	GO:0007275	50
multicellular organismal process	GO:0032501	50
blood vessel development	GO:0001568	5
**biological regulation**	GO:0065007	46
**diverse Regulations**		
**signaling pathway**	GO:0023033	33
**signal transduction**	GO:0007165	25
**cell surface receptor linked signaling pathway**	GO:0007166	0
**biological adhesion**	GO:0022610	22
**cell adhesion**	GO:0007155	22
**cell proliferation**	GO:0008283	13
**Under-represented**		
**cellular metabolic process**	GO:0044237	38
**gene expression**	GO:0010467	14
**nucleic acid metabolic process**	GO:0090304	13
**cellular macromolecule biosynthetic process**	GO:0034645	10

The table shows the ORA (parameters: p-value threshold. t_p = 0.05 and Bonferroni α correction) for genes, which were less upregulated in EAE mice treated with R-flurbiprofen as compared to vehicle treated mice. The functional areas i.e. the "headlines" describing the functions of the set of genes in a comprehensive manner (1) are shown in bold letters aligned left. Further significant GO terms located in the knowledge representation graph (see Suppl. Figure 1) down along the hierarchy are inserted below the respective functional areas. Significant GO terms are a result of over-representation analysis (ORA) of the genes that were less upregulated in EAE mice treated with R-flurbiprofen as compared to vehicle treated mice. The precise definition of the GO terms can be obtained using the AmiGO search tool for GO at *http://amigo.geneontology.org/*. Remarkableness of a term is the product of (i) the certainty that the term describes the gene set and (i) the information of the particular term as describing a subset of genes (1).

1. Lotsch, J., Schaeffeler, E., Mittelbronn, M., Winter, S., Gudziol, V., Schwarzacher, S.W., Hummel, T., Doehring, A., Schwab, M., and Ultsch, A. 2013. Functional genomics suggest neurogenesis in the adult human olfactory bulb. *Brain Struct Funct*.

Legend to Suppl. Figure 1

Graphical representation of the gene ontology (GO) classification showing the polyhierarchy of functional annotations (GO terms) assigned to those genes that were less upregulated in EAE mice treated with R-flurbiprofen as compared to vehicle treated mice.
